# Reduced coupling between cerebrospinal fluid flow and global brain activity is linked to Alzheimer disease–related pathology

**DOI:** 10.1371/journal.pbio.3001233

**Published:** 2021-06-01

**Authors:** Feng Han, Jing Chen, Aaron Belkin-Rosen, Yameng Gu, Liying Luo, Orfeu M. Buxton, Xiao Liu, the Alzheimer’s Disease Neuroimaging Initiative

**Affiliations:** 1 Department of Biomedical Engineering, The Pennsylvania State University, University Park, Pennsylvania, United States of America; 2 Department of Sociology & Criminology, The Pennsylvania State University, University Park, Pennsylvania, United States of America; 3 Population Research Institute, The Pennsylvania State University, University Park, Pennsylvania, United States of America; 4 Department of Biobehavioral Health, The Pennsylvania State University, University Park, Pennsylvania, United States of America; 5 Institute for Computational and Data Sciences, The Pennsylvania State University, University Park, Pennsylvania, United States of America; University of Rochester Medical Center, UNITED STATES

## Abstract

The glymphatic system plays an important role in clearing the amyloid-β (Aβ) and tau proteins that are closely linked to Alzheimer disease (AD) pathology. Glymphatic clearance, as well as Aβ accumulation, is highly dependent on sleep, but the sleep-dependent driving forces behind cerebrospinal fluid (CSF) movements essential to the glymphatic flux remain largely unclear. Recent studies have reported that widespread, high-amplitude spontaneous brain activations in the drowsy state and during sleep, which are shown as large global signal peaks in resting-state functional magnetic resonance imaging (rsfMRI), are coupled with CSF movements, suggesting their potential link to glymphatic flux and metabolite clearance. By analyzing multimodal data from the Alzheimer’s Disease Neuroimaging Initiative (ADNI) project, here we showed that the coupling between the global fMRI signal and CSF influx is correlated with AD-related pathology, including various risk factors for AD, the severity of AD-related diseases, the cortical Aβ level, and cognitive decline over a 2-year follow-up. These results provide critical initial evidence for involvement of sleep-dependent global brain activity, as well as the associated physiological modulations, in the clearance of AD-related brain waste.

## Abstract 2

This study reveals strong coupling between the global fMRI signal and cerebrospinal fluid influx, finding that this is correlated with Alzheimer’s disease-related pathology, disease severity, and cognitive decline. This supports a link between spontaneous low-frequency brain dynamics and Alzheimer’s disease pathology, presumably due to their role in glymphatic clearance.

## Introduction

The pathogenesis of Alzheimer disease (AD) is widely believed to be driven by the aggregation of toxic proteins, e.g., the amyloid-β (Aβ) and tau, which cannot be adequately cleared from the brain [1–3]. The “glymphatic system” plays an important role in the clearance of the toxic proteins in the extracellular interstitial space [4–8]. In this clearance pathway, the movement of the cerebrospinal fluid (CSF) from the periarterial space into the interstitial space, which is facilitated by astroglial aquaporin-4 (AQP4) channels, drives convective interstitial fluid (ISF) flux and interstitial solutes, including Aβ and tau, into the perivenous space surrounding deep-draining veins, from where the effluxed wastes can then be absorbed into the cervical lymphatic system [7,8]. An intriguing finding regarding this glymphatic pathway is that the CSF influx can increase 20-fold during sleep compared with the awake condition [4]. This highly sleep-dependent clearance may contribute to the diurnal fluctuation of interstitial Aβ observed in both humans and mice [9,10], as well as the link between AD pathology and sleep [11–13]. However, this strong sleep dependency obscured our understanding of the driving forces behind the CSF influx of the glymphatic process. Arterial pulsation [6,14–16] and respiration [17,18], which have been hypothesized to be the major drivers of glymphatic CSF movements, may not sufficiently explain this sleep-dependent enhancement since they remain similar or even decrease during sleep due to reduced physical activities [4,19–23].

The glymphatic process may be related to sleep-dependent global brain activity recently observed with resting-state functional magnetic resonance imaging (rsfMRI) [24–26]. A pioneering study using fast fMRI technique is the first one pointing out a potential relationship between low-frequency (<0.1 Hz) rsfMRI signals and the glymphatic system [27]. The most recent study using the same technique showed an increase of brain signals of the cardiac and respiratory frequencies during sleep, but this increase is considerably smaller than that of the low-frequency range (<0.1 Hz) [28]. This sleep dependency of low-frequency rsfMRI signals is likely caused by characteristic global brain activity captured by the mean rsfMRI signal, which we will refer to as the global blood oxygen level–dependent (BOLD) signal hereafter. Converging evidence has shown the neural origin of the global BOLD signal [26,29] and its strong dependency on brain arousal state [29,30], particularly the light sleep stage [31–36]. Moreover, caffeine [37] can effectively suppress this global signal component, whereas hypnotic drugs, i.e., midazolam and zolpidem [38–40], and sleep deprivation [41] had opposite effects. Not until recently was it found that the global BOLD signal is tightly linked to a specific electrophysiological event of 10 to 20 seconds that represents the transient arousal modulation during the drowsy state or sleep [25,26]. Importantly, the large global BOLD changes are also accompanied by slow (<0.1 Hz) but strong modulations in cardiac [42] and respiratory [43,44] activities, which could drive the CSF flow [6,14–18,45], and also in vessel tone [46], which has been directly linked to cortical Aβ clearance in recent animal work [47]. Consistent with this idea, the large global BOLD signal fluctuation was found to be accompanied by CSF inflows during sleep in humans [48]. All these findings suggested the potential role of the global BOLD signal and associated physiological modulations in the glymphatic process important for AD pathogenesis. Therefore, we hypothesize that the coupling between the global BOLD signal and CSF inflow is related to AD-related pathology.

To test this hypothesis, we examine multimodal data from the Alzheimer’s Disease Neuroimaging Initiative (ADNI) [3] with a focus on the coupling of the global BOLD signal and CSF signal and its relationship with AD-related neurobiological and neuropsychological measures. We found a strong coupling between the global brain signal and CSF signal, which is significantly attenuated in the AD-related disease groups and correlated with multiple AD risk factors. The BOLD–CSF coupling strength is significantly correlated with the cortical Aβ level and also predicts the cognitive decline in the subsequent 2 years. The findings suggest an important role for the neural and physiological processes associated with the global brain BOLD signal in the AD pathogenesis.

## Results

### Sample characteristics

We used imaging and behavioral data from 118 participants in the ADNI project. The sample included 7 AD patients, 62 mild cognitive impairment (MCI) patients, 18 significant memory concern (SMC) patients, and 31 healthy controls (HCs) (see Table 1 for a detailed sample characteristics). They were selected based on the availability of rsfMRI, florbetapir positron emission tomography (PET) measurement of Aβ, and behavioral measures through Mini-Mental State Examination (MMSE). The 118 participants underwent 158 rsfMRI sessions that are associated with the baseline and follow-up (after approximately 2 years) Aβ-PET and MMSE measurements (see Materials and methods for details about participant selection). This sample allowed us to link the baseline rsfMRI data to longitudinal changes of AD-related markers. Data from 29 out of 118 participants were collected in 3 consecutive time points (i.e., 3 Aβ-PET and MMSE sessions at year 0, 2, and 4 and corresponding rsfMRI sessions at year 0 and 2) and were then split into 2 pairs of measurements by reusing the middle time point as the baseline of the third time point. Similarly, we split the data of 4 participants with 4 consecutive measurements and 1 participant with 5 consecutive measurements to increase the pairs of measures with a 2-year interval.

**Table 1:** Participant baseline characteristics.

ADNI (*N* = 118)		(A) AD (*N* = 7)	(B) MCI (*N* = 62)	(C) SMC (*N* = 18)	(D) HC (*N* = 31)	*p*-value
Age at baseline (M/SD)		79.53 (4.55)	72.72 (7.17)	73.17 (5.49)	74.46 (5.78)	**A and B = 0.02**; **A–C = 0.01** **A–D = 0.04**; B and C = 0.80 B–D = 0.24; C and D = 0.45
Gender (M/F)		2/5	32/30	9/9	15/16	A and B = 0.43; A–C = 0.41 A–D = 0.43; B and C = 1.00 B–D = 0.83; C and D = 1.00
APOE ε4 status (Neg/Pos) (5 N/A)		0/7	33/27 (2 N/A)	13/5	19/9 (3 N/A)	**A and B = 0.01**; **A–C = 0.002** **A–D = 0.002**; B and C = 0.28 B–D = 0.35; C and D = 1.00
MMSE (M/SD)	First visit	22.00 (2.38)	27.95 (1.95)	29.17 (0.86)	28.77 (1.26)	**A and B <0.001**; **A–C <0.001** **A–D <0.001**; **B and C = 0.01** **B–D = 0.04**; C and D = 0.25
	24 mo follow-up	18.14 (5.79)	27.08 (3.50)	28.94 (1.00)	28.74 (1.91)	**A and B <0.001**; **A–C <0.001** **A–D <0.001**; **B and C = 0.03** **B–D = 0.02**; C and D = 0.68
Aβ florbetapir SUVR (M/SD)	First visit	1.08 (0.07)	0.90 (0.14)	0.83 (0.10)	0.82 (0.13)	**A and B = 0.001; A–C <0.001** **A–D <0.001**; B and C = 0.08 **B–D = 0.008**; C and D = 0.61
	24 mo follow-up	1.11 (0.06)	0.91 (0.15)	0.84 (0.12)	0.83 (0.13)	**A and B <0.001**; **A–C <0.001** **A–D <0.001**; B and C = 0.08 **B–D = 0.01**; C and D = 0.77

*p*-Values are derived from 2-sample *t* test for continuous measures and from Fisher exact test for categorical measures. The data underlying this table can be found in S1 Data.

24 mo follow-up, 24 months follow-up; Aβ, amyloid-β; Aβ florbetapir SUVR, the whole cortical amyloid beta from PET AV45 analysis normalized composite reference region; AD, Alzheimer disease participants; ADNI, Alzheimer’s Disease Neuroimaging Initiative; APOE ε4 status (Neg/Pos), not APOE ε4 carrier/APOE ε4 carrier; HC, healthy control; M/F, male/female; M/SD, mean/standard deviation; MCI, mild cognition impairment; MMSE: Mini-Mental State Examination; SMC, significant memory concern.

### A coupling between the global BOLD signal and CSF signal

**Fig 1:**
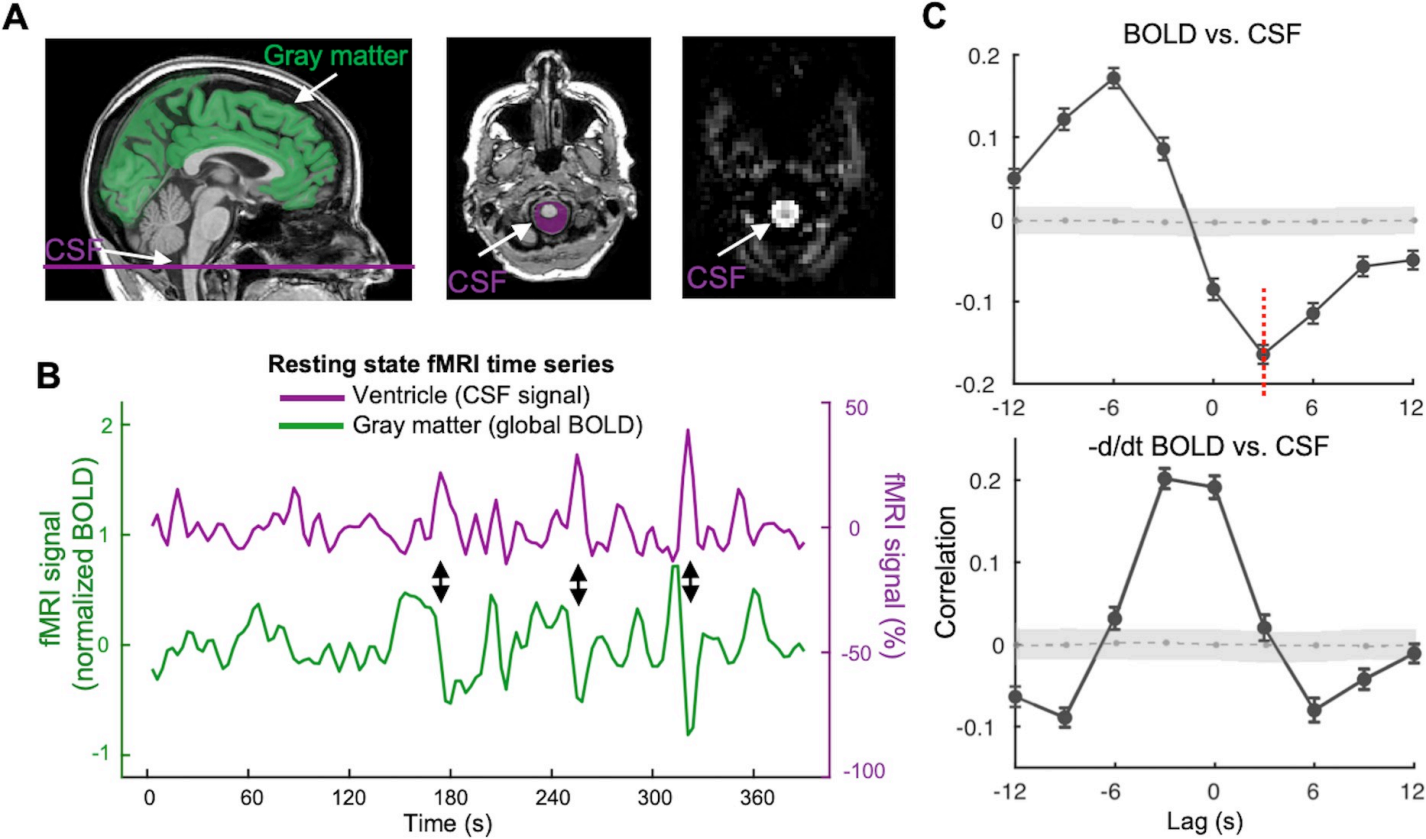
The global BOLD signal is coupled with CSF changes. (**A**) The global BOLD signal was averaged across the gray matter regions (the green mask on an exemplary T1-weighted image in the left panel), whereas the CSF signal was extracted from the CSF regions at the bottom slice of the fMRI acquisition (the middle and right panel). The CSF appears much brighter than the surrounding areas in the T2*-weighted fMRI image (the right panel). (**B**) The global BOLD signal and the CSF signal from a representative participant showed corresponding changes (indicated by black arrows). We also included a different version of the global BOLD signal in percentage changes to show the amplitude of signal fluctuation (**S12A Fig**). (**C**) The cross-correlation function between the global BOLD signal and the CSF signal averaged across 158 sessions (upper) and the one between the negative derivative of global BOLD signal and the CSF signal (lower). The gray shaded region denotes 95% confidence intervals calculated with shuffled signals (see Materials and methods for details; see **S12B Fig** for all the 158 cases and their standard deviation (SD) of the BOLD–CSF cross-correlation function). Gray dashed lines in the shaded region shows mean correlation of the null distribution from permutation test at each time lag. Error bar in this figure represents the SEM. These cross-correlation functions show a very similar shape to those reported in the previous study [48]. The cross-correlation (−0.17, *p* < 0.0001, permutation test) at the +3-second lag (red dashed line), which also showed the strongest coupling in the previous study [48], was used for quantifying the BOLD–CSF coupling for subsequent analyses. The data underlying this figure can be found in S1 Data. BOLD, blood oxygen level–dependent; CSF, cerebrospinal fluid; fMRI, functional magnetic resonance imaging; SEM, standard error of the mean.

We first examined whether the BOLD–CSF coupling described in the previous human study [48] is also present in the ADNI rsfMRI data collected in relatively older participants and with lower spatial and temporal resolutions. Similar to the previous study, we extracted CSF signals from the bottom slice of rsfMRI acquisition, which is around the bottom of the cerebellum, to maximize sensitivity to the CSF inflow effect [48]. The CSF regions can be easily identified in the T2*-weighted fMRI images since they appear much brighter than surrounding tissues (the right panel in Fig 1A, corresponding to the purple mask in the middle panel). Meanwhile, we obtained the global BOLD signal by extracting and averaging BOLD signals from the whole-brain gray matter regions (Fig 1A, the green mask). As shown in a representative participant (Fig 1B), large modulations in the global BOLD signal are often accompanied by corresponding large changes in the CSF signal, suggesting a coupling between the two. To better quantify this BOLD–CSF coupling, we calculated the cross-correlation function between the two, which showed their Pearson correlations with different time lags. The resulting BOLD–CSF cross-correlation function is characterized by a positive peak (0.17; *p* < 0.0001, permutation test; see Materials and methods for details) at the lag of −6 seconds (i.e., shifting CSF ahead of time by 6 seconds) and a negative peak (*r* = −0.17; *p* < 0.0001, permutation test) at the lag of +3 seconds (Fig 1C, upper panel); intriguingly, this temporal pattern closely resembles the one obtained in the previous study [48] despite with the lower temporal resolution. The cross-correlation function was also calculated between the negative derivative of the global BOLD signal and the CSF signal. It showed a strong positive peak around the lag of −3 second (0.20; *p* < 0.0001, permutation test), also consistent with the previous finding [48]. Taken together, these results confirmed the existence of a significant BOLD–CSF coupling with specific and reproducible temporal patterns in the ADNI dataset.

### Relationships between the BOLD–CSF coupling and AD-related pathology

**Fig 2:**
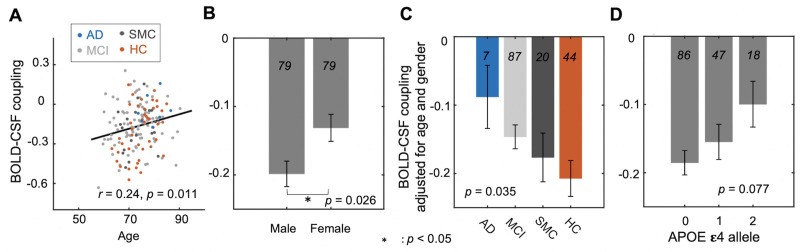
The dependency of the BOLD–CSF coupling on AD risk factors and disease conditions. (**A**) The strength of the BOLD–CSF coupling, quantified as the correlation between the global BOLD signal and CSF at +3-second lag, shows a significant correlation (Spearman’s *r* = 0.24, *p* = 0.011, the linear mixed model with Satterthwaite method) with age across the 158 sessions. The linear regression line was estimated based on the linear least-squares fitting [49]. (**B**) Male participants showed a larger amplitude of the BOLD–CSF coupling as compared with females (*p* = 0.026). (**C**) The BOLD–CSF coupling, after adjusting for age and gender, decreases gradually (*p* = 0.035) from the HCs, to SMC, to the MCI, and then to AD group. (**D**) The age- and gender-adjusted BOLD–CSF coupling is also marginally (*p* = 0.077) correlated with the APOE ε4 allele. Error bar in this figure represents the SEM. The sample sizes of each subgroups are shown by numbers on the bars of the bar plots. The data underlying this figure can be found in S1 Data. The analysis was repeated for an augmented sample with more AD patients and HCs; see **S1 Fig** for the results. AD, Alzheimer disease; APOE, apolipoprotein E; BOLD, blood oxygen level–dependent; CSF, cerebrospinal fluid; HC, healthy control; MCI, mild cognitive impairment; SEM, standard error of the mean; SMC, significant memory concern.

We then investigated whether the BOLD–CSF coupling is related to the AD-related pathology, including AD risk factors, disease condition, and neurobiological and neuropsychological markers. We used the negative peak BOLD–CSF correlation at the 3-second lag (Fig 1C, the red dashed line in the upper panel) to represent the strength of the BOLD–CSF coupling, since the previous study with superior imaging quality found the strongest BOLD–CSF correlation at the negative peak around the same lag [48]. The BOLD–CSF coupling strength was first related to the age and gender, 2 major risk factors for AD. It is significantly correlated with age (Spearman’s *r* = 0.24; *p* = 0.011 based on a linear mixed effect model with Satterthwaite method, the same hereinafter unless noted otherwise; see Materials and methods for details) with older participants showing relatively weaker (less negative) BOLD–CSF coupling (Fig 2A). The BOLD–CSF coupling also appeared to be significantly stronger (*p* = 0.026) in males than in females (Fig 2B). We then compared the BOLD–CSF coupling in participants under different disease conditions after adjusting for age and gender. The BOLD–CSF coupling strength exhibited a dose–response relationship with severity of disease condition, lower (*p* = 0.035) from the HC > SMC > MCI > AD groups (Fig 2C). The age- and gender-adjusted BOLD–CSF coupling was also compared across different apolipoprotein E (APOE) gene carriers and showed a marginally significant (*p* = 0.077) dependency on the copies of APOE ε4 alleles (Fig 2D). The results regarding the disease condition and APOE gene could be significantly underpowered due to limited AD patients (*N* = 7) and 2 APOE ε4 alleles carriers (*N* = 11). We thus augmented the sample by adding 22 AD and 14 HC participants, 5 of whom carry 2 APOE ε4 alleles, from another subset of the ADNI dataset (see Materials and methods section for details about data selection). The same analysis on this augmented sample (S1 Fig; see the sample characteristics in S1 Table) led to similar findings with improved statistical significance, particularly for the difference (*p* = 0.0078) between the AD and HC groups (S1C Fig). The relationship between the BOLD–CSF coupling and the APOE ε4 copies also reached the statistical significance (*p* = 0.041) with the augmented sample size (S1D Fig). It is also worth noting that the relationships of the BOLD–CSF coupling to age (*p* = 0.020) and gender (*p* = 0.032) remained significant after controlling for disease condition (S2 Fig). In summary, the BOLD–CSF coupling was gradually weaker from the HC to SMC, to MCI, and then to AD groups, conditions known to show a gradually increasing severity of AD-related symptoms; it also appeared to be weaker in older participants, females, and APOE ε4 gene carriers, who are known to have a higher risk of developing AD pathology.

**Fig 3:**
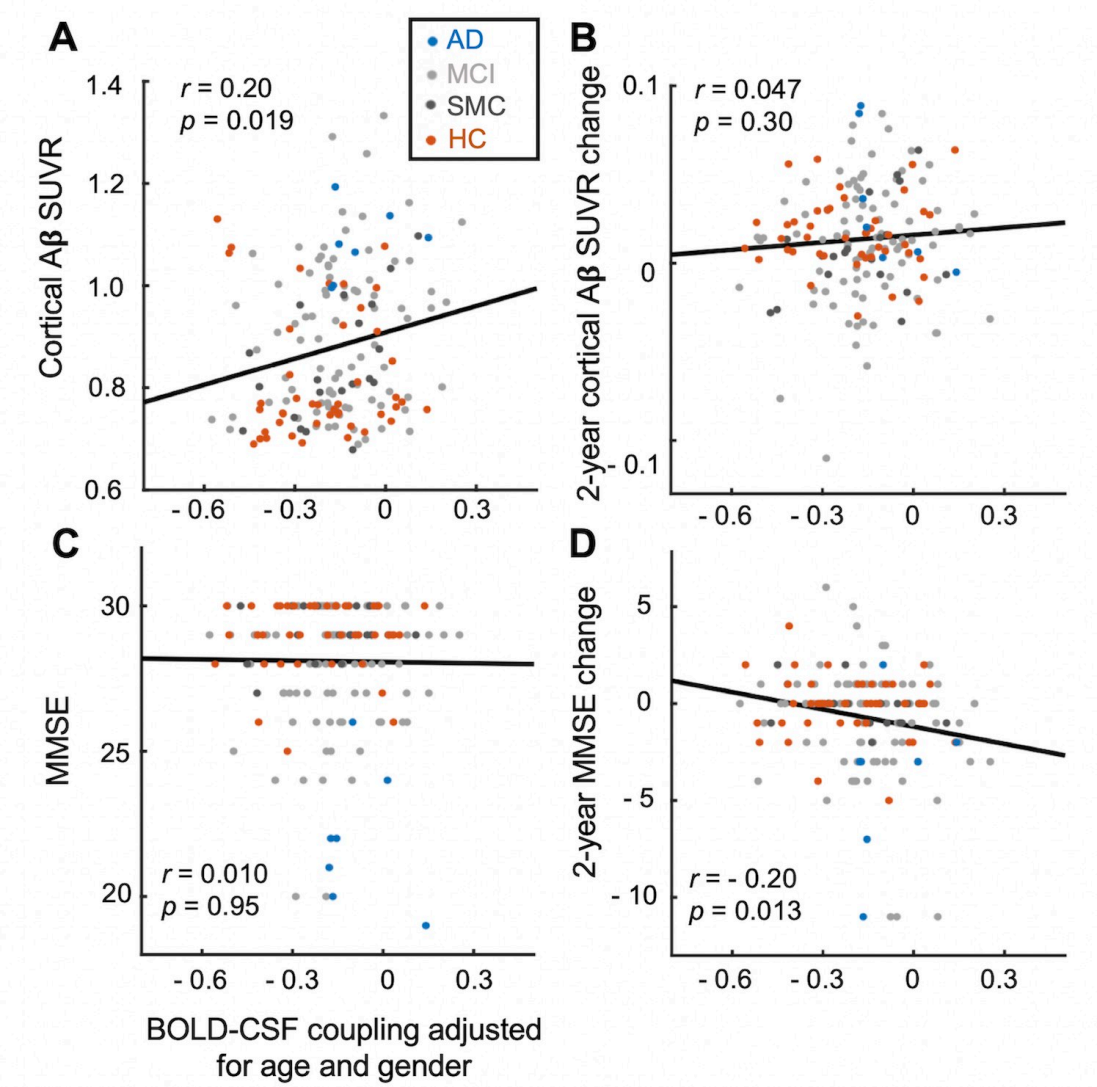
The BOLD–CSF coupling is correlated with the cortical Aβ and cognitive decline. (**A**, **B**) The BOLD–CSF coupling adjusted for age and gender is significantly correlated (Spearman’s *r* = 0.20, *p* = 0.019, *N* = 158, the linear mixed model with Satterthwaite method) with the cortical Aβ SUVRs at baseline (**A**) but not their changes in the following 2 years (**B**). (**C**, **D**) The BOLD–CSF coupling adjusted for age and gender is significantly correlated (Spearman’s *r* = −0.20, *p* = 0.013, *N* = 158) with the MMSE score changes in the following 2 years (**D**) but not with its baseline value (**C**). Each dot represents a single session. AD, MCI, SMC, and HC sessions are colored with blue, light gray, dark gray, and orange, respectively. The data underlying this figure can be found in S1 Data. Aβ, amyloid-β; AD, Alzheimer disease; BOLD, blood oxygen level–dependent; CSF, cerebrospinal fluid; HC, healthy control; MCI, mild cognitive impairment; MMSE, Mini-Mental State Examination; SMC, significant memory concern; SUVR, standardized uptake value ratio.

The BOLD–CSF coupling was then correlated with neurobiological and neuropsychological markers more specifically related to AD pathology, including the cortical Aβ level as measured by the florbetapir standardized uptake value ratio (SUVR) averaged over the entire cortex [50] and the cognitive performance as measured by the MMSE score. After adjusting for age and gender, a significant positive correlation (Spearman’s *r* = 0.20, *p* = 0.019, the linear mixed model with Satterthwaite method) was found between the BOLD–CSF coupling and the cortical Aβ level measured at baseline around the same time (Fig 3A). Thus, participants showing weaker BOLD–CSF coupling exhibited more Aβ accumulated in the cortex. We did not, however, find a significant relationship between the BOLD–CSF coupling and the Aβ accumulation in the subsequent 2 years (Fig 3B). Rather, the 2-year longitudinal change of the MMSE score (Fig 3D), but not its baseline value (Fig 3C), is negatively correlated (Spearman’s *r* = −0.20, *p* = 0.013) with the negative BOLD–CSF coupling, evidence for an association between weak BOLD–CSF coupling and large cognitive decline over the following 2 years. These associations remained significant (*p* < 0.05) even when excluding the AD and HC groups from the analysis (S3 Fig), using another reference region (i.e., eroded white matter) for Aβ SUVR calculation (S4 Fig), conducting a subject-based analysis that only used 1 pair of sessions for each participant (S5 Fig; see Materials and methods section for details), controlling for head motions estimated by the mean framewise displacement (FD) (S6 and S7 Figs), or including the data batch (i.e., ADNI 2, ADNI 3, or ADNI GO) as a covariate of the model (S8 Fig). We also examined how the BOLD–CSF correlations at different time lags are related to the Aβ and cognition decline, and the results showed that they were only significant (*p* < 0.05) at time lags showing large negative BOLD–CSF coupling (i.e., at lag 0 second and +3 seconds; S9 Fig). The relationships of the BOLD–CSF coupling with age, gender, disease condition, and APOE gene were reassessed while controlling for the cortical Aβ level, and the BOLD–CSF coupling remained significantly correlated with age (*p* = 0.049) but not for gender (*p* = 0.06), disease condition (*p* = 0.32), and APOE gene (*p* = 0.86) (S10 Fig).

### The BOLD–CSF coupling’s relationship with AD pathology is not dependent on the global signal amplitude

**Fig 4:**
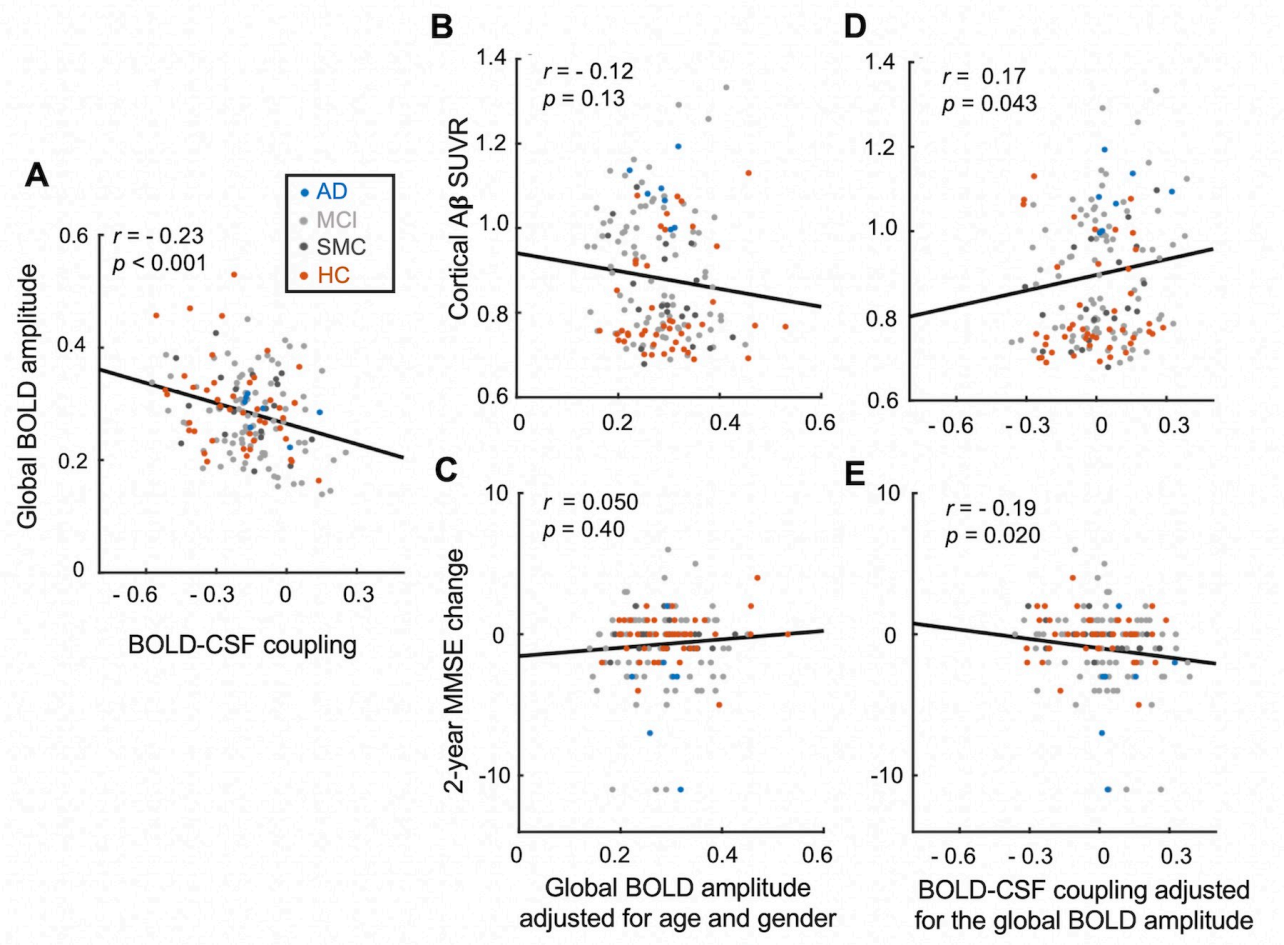
The role of global BOLD amplitude in the associations between BOLD–CSF coupling and AD-related markers. (**A**) The strength of the BOLD–CSF coupling is dependent (Spearman’s *r* = −0.23, *p* < 0.001, *N* = 158 sessions) on the fluctuation amplitude of the global BOLD signal after adjusting for age and gender. (**B**, **C**) The amplitude of the global BOLD signal, adjusted for age and gender, is not significantly correlated (*p* > 0.13) with either the cortical Aβ level (**B**) or the 2-year longitudinal change of MMSE score (**C**). (**D**, **E**) The BOLD–CSF coupling remains significantly correlated (p < 0.05) with the cortical Aβ level (**D**) and the MMSE changes (**E**) after adjusting for age, gender, and global BOLD amplitude. Each dot represents a single session. AD, MCI, SMC, and HC sessions are colored with blue, light gray, dark gray, and orange, respectively. The data underlying this figure can be found in S1 Data. Aβ, amyloid-β; AD, Alzheimer disease; BOLD, blood oxygen level–dependent; CSF, cerebrospinal fluid; HC, healthy control; MCI, mild cognitive impairment; MMSE, Mini-Mental State Examination; SMC, significant memory concern; SUVR, standardized uptake value ratio.

Next, we sought to understand whether the association between the BOLD–CSF coupling and AD pathology is mediated by the change of global BOLD signal amplitude. Converging evidence has suggested that the fluctuation amplitude of global BOLD signal [25,26,29–36,48], as well as its coupling with CSF [48], is highly dependent on sleep/wake state, and much stronger during sleep than wake. Thus, the weak BOLD–CSF coupling may simply reflect a tendency of staying awake inside the scanner for patients with AD-related pathology. To test this possibility, we investigated how the global BOLD amplitude, i.e., the fluctuation amplitude of the global BOLD signal, may affect the relationship between the BOLD–CSF coupling and AD-related markers. Consistent with the previous study [48], a stronger BOLD–CSF coupling tends to be associated with (*p* = 0.00062) a larger global BOLD amplitude (Fig 4A) indicative of a drowsy state or sleep [29,30]. However, the global BOLD amplitude is not significantly correlated with either the baseline cortical Aβ (*p* = 0.13) or the longitudinal MMSE changes (*p* = 0.40) (Fig 4B and 4C). Moreover, after adjusting for the global BOLD amplitude, the relationships of the BOLD–CSF coupling with the baseline Aβ and the longitudinal MMSE changes remained significant (*p* = 0.043 and 0.020, respectively, Fig 4D and 4E, the linear mixed model with Satterthwaite method). We also repeated the above analysis by replacing the global BOLD amplitude with an fMRI-based arousal index [51,52]. Although the arousal index estimates the arousal level through distinct information, i.e., the co-activation patterns of individual fMRI volumes, it showed a strong correlation with the global BOLD amplitude (*r* = 0.50, *p* = 1.55 × 10^−10^, *N* = 158). Similarly, controlling for the effect of the arousal index did not affect the relationship of the BOLD–CSF coupling with either the baseline Aβ (*p* = 0.022) or the longitudinal MMSE changes (*p* = 0.0094) (S11 Fig). Therefore, the weakening of the BOLD–CSF coupling associated with the Aβ accumulation and cognitive decline is unlikely due to a reduced global BOLD amplitude that is suggestive of a brain state change, but reflects a true disruption of the coupling between the global brain signal and CSF flux.

## Discussion

We show a strong coupling between the global brain BOLD signal and CSF flux in the rsfMRI data from older adults in the ADNI project. The strength of this BOLD–CSF coupling varied considerably across participants and is significantly weaker in those with a higher risk for AD or having developed AD-related diseases. Importantly, the BOLD–CSF coupling strength is significantly associated with the cortical Aβ level and the MMSE score change in the subsequent 2 years, indicating its close relationship with the AD pathophysiology. These findings suggest an important role of the neural and physiological modulations associated with the large global brain signal in Aβ clearance and thus the AD pathogenesis, presumably through their involvement in the glymphatic process.

The global brain signal and CSF flow become weakly coupled in participants with AD-related diseases or at higher risk of developing AD. A gradual weakening of the BOLD–CSF coupling is evident from the HC, to SMC, to MCI, and then to AD groups demonstrating increasing severity of AD-related symptoms. The BOLD–CSF coupling also appeared significantly weaker in older participants and females, who are known to have a higher risk of developing AD [53,54], and the differences remain significant after controlling for the disease condition. The BOLD–CSF coupling is also weaker in APOE gene carriers, but this association became nonsignificant after adjusting for disease condition. The dependency of the BOLD–CSF coupling on the disease condition, as well as the APOE gene, can be at least in part attributed to its dependency on cortical Aβ, because they are no longer significant when controlling for Aβ levels. The association between the BOLD–CSF coupling and the longitudinal MMSE change may also be related to the cortical Aβ accumulation, which appears earlier than the cognitive decline in the AD pathogenesis [55,56]. Therefore, the key to understanding the link of BOLD–CSF coupling with the AD-related pathology lies in its relationship with the cortical Aβ level, which is likely related to the Aβ clearance through the glymphatic system.

The BOLD–CSF coupling could be related to the characteristic global brain activity at transient arousal modulations. Despite the lower spatial and temporal resolutions than the previous study [48], the ADNI data displayed a consistent BOLD–CSF coupling as evidenced by the same pattern of the cross-correlation functions [48]. The BOLD–CSF coupling has been shown to be present during sleep with the global BOLD signal showing large-amplitude fluctuations [48]. Consistent with this finding, we observed stronger BOLD–CSF coupling at larger global signal amplitudes (Fig 4A). The dependency of the global BOLD signal on the brain state, particularly its large fluctuations during light sleep stages [32], has been repeatedly reported [29,31–41]. However, not until recently did we have more clues about the neurophysiological underpinnings of this phenomenon. A recent multimodal study on monkeys and humans suggested that the large-amplitude global BOLD fluctuation is driven by global brain co-activations that are associated with a characteristic neurophysiological event, i.e., the sequential spectral transition (SST) event [26]. Importantly, both the spatial pattern of the global co-activations, which showed specific deactivations in subcortical arousal-regulating regions [26], and the time–frequency pattern of the SST, which displayed a transition from wake-dominant alpha-beta (8 to 30 Hz) activity to sleep-dominant delta (0.5 to 4 Hz) activity [25,26], indicated a transient modulation in brain arousal. Thus, the large global BOLD signal, as well as its coupling with CSF, could be related to transient arousal modulations in the drowsy state or during sleep.

The arousal-related global brain co-activations are accompanied by physiological modulations that can potentially drive CSF movements with the involvement of the autonomic system. First, the arousal-related global brain activity is likely associated with slow (<0.1 Hz) but large arterial constrictions/dilations that may drive CSF flow. The arterial pulsation has been regarded as the major driving force that propels CSF flow along the periarterial spaces [6,14–16]. Despite the cardiac function decreases during sleep [19,21,22], their potential effect on glymphatic function may still increase with reduced tissue resistance due to increased interstitial spaces [4]. This idea is supported by an increase in the amplitude of brain signals of cardiac and respiratory frequencies during sleep [28]. However, the increase of these fast brain signals is much smaller than that of the low-frequency (<0.1 Hz) rsfMRI signals [28]. The finding should not be regarded as evidence against the role of arterial pulsations in glymphatic function. In fact, the low-frequency rsfMRI signals are likely linked to spontaneous low-frequency modulations of arterial pulsations. The global BOLD signal was correlated with slow components of peripheral cardiac signals, including envelop amplitude and heart rate variability [57–60], and also preceded by strong, anti-phase BOLD changes in the internal carotid arteries (ICAs) [61]. Given the saturated oxygenation in arteries, the ICA signal changes could be attributed to cerebral blood volume changes reflecting strong arterial constrictions/dilations. The global BOLD component also showed a systematic delay from the gray matter ventromedially toward deep white matter structures, which was hypothesized to be driven by the modulation of pial arteries through sympathetic innervation from the superior cervical ganglion [42]. Overall, the global BOLD signal is tightly linked to, and may actually drive, spontaneous low-frequency modulation of arterial pulsations, which has been convincingly linked to glymphatic clearance of Aβ [47]. Second, the global BOLD signal is also accompanied by strong low-frequency (<0.1 Hz) respiratory modulations [44,62], presumably through the same autonomic regulation at the transient arousal modulations. Respiration is widely regarded as another major driver of the glymphatic CSF flow [17,18]. Inspiration was particularly identified as the major driving force [63] and can induce, through a reduction of intrathoracic pressure, an upward CSF flow [63] that would produce inflow effect as observed in the current study. Altogether, the low-frequency global brain activity captured by the global BOLD signal may modulate, via the autonomic system, arterial pulsation and respiration, which, in turn, drive CSF flows of low frequency. It remains a challenge for future studies to verify this hypothesis or find other mechanisms underlying the BOLD–CSF coupling. Despite the accumulated evidence linking the BOLD–CSF coupling to the arousal-related global neural activity, the current study cannot exclude, due to a lack of electrophysiological recordings, the possibility that the BOLD–CSF coupling originated purely from other physiological processes unrelated to neural activity.

The neural and physiological changes associated with the global BOLD signal may further facilitate the glymphatic clearance in 2 hypothetical ways. First, they might facilitate CSF flow by affecting its production. The superior cervical ganglia are known to have strong sympathetic inhibitory control over the choroid plexuses in the brain ventricles [64], which are the major brain structure to produce CSF. The autonomic modulations associated with the global brain co-activations are thus expected to also affect the CSF production that may, in turn, affect its movement. Second, the coordinated CSF movement and global brain co-activations might be another factor facilitating brain waste clearance. The arousal-related global brain activity showed a much larger amplitude in the motor/sensory regions [26], where Aβ accumulates much slower than the other higher-order cortical regions in the AD prognosis [65], suggesting a potential interaction effect between brain activation and the glymphatic process. However, these 2 potential mechanisms are purely conjectural. They remain to be investigated by future studies, particularly those on animal models.

The co-modulation of the BOLD–CSF coupling and the cortical Aβ should not be attributed to the change of global BOLD signal amplitude suggestive of the brain state change, nor interpreted as a causal relationship. The BOLD–CSF coupling is significantly correlated with the global BOLD signal amplitude, as well as the fMRI-based arousal index, confirming its dependency on the brain state. However, the global signal amplitude itself is not correlated with the cortical Aβ level, nor did controlling for this factor affect the association between the BOLD–CSF coupling and Aβ. Thus, the reduced BOLD–CSF coupling at the high Aβ level likely reflects a disrupted coupling between the global brain activity and CSF movements. At the same time, this correlation alone cannot tell us whether the disrupted BOLD–CSF coupling is a cause or a consequence of the Aβ accumulation. It is worth noting that the BOLD–CSF coupling is correlated with age, even controlling for cortical Aβ level. However, this piece of evidence is insufficient to exclude the possibility that the increased cortical Aβ, which is often deposited in the perivascular spaces to result in cerebral amyloid angiopathy [66,67], may block CSF flux coupled with the global brain activity and thus disrupt the BOLD–CSF coupling. It is also possible that the decrease of BOLD–CSF coupling and the accumulation of cortical Aβ are 2 processes facilitating each other after either one initiating their interaction. Testing these possibilities remains a challenge for future work.

There are a few limitations for the current study. First, the fMRI-based quantifications, i.e., the arousal index and the fluctuation amplitude of the global BOLD signal, were used to estimate the arousal state of participants. Although similar arousal metrics have been verified by studies using simultaneous recordings of fMRI and electrophysiology [51,68], arousal measures using other modalities, such as electroencephalography (EEG) vigilance index [68], would provide a more independent estimation of brain arousal state. Moreover, the EEG vigilance measures would not be confounded by potential changes of neurovascular coupling in old and cognitive impaired patients, compared with those hemodynamic vigilance markers. Second, the conventional fMRI technique used by ADNI would not allow us to sample fast brain signals of the cardiac and respiratory frequencies. It would be interesting to employ fast fMRI approaches [48] to measure BOLD–CSF coupling of the fast frequencies and then to compare with the low-frequency BOLD–CSF coupling in terms of their association with AD pathology. Third, the global BOLD signal may still contain CSF signals due to the limited spatial resolution of fMRI and partial volume effect. However, this contribution is deemed negligible because (1) the CSF deep inside the imaging volume would have very small inflow effect [48]; (2) the CSF areas are much smaller than the gray matter regions; and (3) the potential CSF contamination is expected to contribute mostly to the zero-lag BOLD–CSF correlation, whereas we used the correlation at the time lag of +3 seconds to measure the BOLD–CSF coupling.

In summary, we found a strong coupling between the global BOLD signal and the CSF inflow in the ADNI rsfMRI data. More importantly, the strength of this BOLD–CSF coupling was significantly associated with various factors related to AD pathology, including the cortical Aβ level. These findings provide initial evidence for the role of large-scale neural and physiological modulations, which are associated with transient arousal modulations in the drowsy state or during sleep, in the glymphatic clearance of toxic brain wastes, including Aβ. They also suggest that the coupling of the global brain signal and CSF inflow may serve as a potential imaging marker for clinical evaluation.

## Materials and methods

### Participants and study data

This is an analytic observational study to investigate the potential relationship between the coupling between the global brain activity and CSF inflow and the AD-related pathology. We included 118 participants from the ADNI project (ADNI-GO, ADNI-2, and ADNI-3) based on the availability of rsfMRI, longitudinal MMSE scores, and longitudinal 18F-AV45 amyloid PET. Specifically, we selected the participants who had rsfMRI at baseline and amyloid PET and MMSE data at baseline and in a 2-year follow-up. The group include AD (*N* = 7), MCI (early MCI and late MCI included; *N* = 62), SMC (*N* = 18), and HC (*N* = 31) participants as defined by ADNI (http://adni.loni.usc.edu/study-design/)), and no participants in the present study experienced changes in the disease condition over the 2-year period. Among them, 113 participants also have available APOE genotype information. Table 1 summarized the participant characteristics, including age at baseline, gender, and numbers of APOE ε4 allele carrying as well as corresponding longitudinal MMSE scores and PET-SUVRs. Since the sample size determined by the above inclusion criteria was too small for the AD group, we also augmented the data by another subset of ADNI data used for a different research project. This second subset of ADNI data includes participants with longitudinal rsfMRI data at 2 time points separated by 2 years, but not all of them had the corresponding longitudinal amyloid PET or MMSE data. A total of 23 AD and 18 HC sessions from 22 AD and 14 HC participants were added into the original sample to create an augmented sample (see S1 Table for the participant characteristics of the augmented sample), which was only used for reexamining the effects of age, gender, disease condition, and APOE gene on the BOLD–CSF coupling. All participants provided written informed consent, and investigators at each ADNI participating sites obtained ethical approval from the corresponding institutional review board (IRB; http://adni.loni.usc.edu/wp-content/uploads/2013/09/DOD-ADNI-IRB-Approved-Final-protocol-08072012.pdf). ADNI data were collected in accordance with the principles of the Declaration of Helsinki.

The rsfMRI, MMSE, APOE genotype, and amyloid PET data at baseline were obtained in the same study visit (the “visit” was defined by ADNI; see “visit code” details in https://adni.loni.usc.edu/wp-content/uploads/2008/07/inst_about_data.pdf). The MMSE and amyloid PET data acquired in a 2-year follow-up (25.0 ± 1.5 months interval for the MMSE interval and 23.9 ± 1.2 months interval for the amyloid PET) were also used. Amyloid PET SUVR data, MMSE scores, and APOE gene genotypes were directly obtained from “UC Berkeley—AV45 Analysis [ADNI1,GO,2,3] (version: 07_28_19)” [69,70], “Mini-Mental State Examination (MMSE) [ADNI1,GO,2,3]” [71], and “APOE—Results [ADNI1,GO,2,3],” respectively, from the ADNI website (http://adni.loni.usc.edu/). There are a total of 158 sessions from the 118 participants having the baseline rsfMRI and 2-year longitudinal MMSE and amyloid PET data. A total of 29 participants have 2 rsfMRI sessions at years 0 and 2, as well as the MMSE and amyloid PET at years 0, 2, and 4; 4 participants with 3 rsfMRI sessions (for each participant) with corresponding 4 sessions of MMSE and amyloid PET (2-year interval); and 1 participant with 4 fMRI sessions and 5 corresponding MMSE and amyloid PET sessions. Overall, our data included 7 sessions from 7 AD patients, 87 sessions from 62 MCI patients, 20 sessions from 18 SMC patients, and 44 sessions from 31 HC participants. The APOE genotype data are available for 151 sessions from 113 participants.

The use of de-identified data from the ADNI and the sharing of analysis results have been reviewed and approved by the Pennsylvania State University IRB (IRB#: STUDY00014669) and also strictly followed the ADNI data use agreements.

### Image acquisition and preprocessing

All rsfMRI data were acquired at 3 Tesla MR scanners in different ADNI participating sites following the same protocol (recent models of MR scanners from General Electric (GE, Chicago, United States) Healthcare, Philips Medical Systems (Philips, Amsterdam, Netherlands), and Siemens Medical Solutions (Siemens, Erlangen, Germany) were used to collect the MRI data at multiple sites, see details at: http://adni.loni.usc.edu/methods/documents/mri-protocols/). Each imaging session began with an MPRAGE sequence (echo time (TE) = 3.1 ms, repetition time (TR) = 2,300 ms), which was used for anatomical segmentation and template normalization [72] (see acquisition details in http://adni.loni.usc.edu/methods/documents/). For rsfMRI acquisition, 140 (ADNI-GO and ADNI-2) or 200 (ADNI-2 (extended fMRI sessions) and ADNI-3) fMRI volumes were collected with an echo-planar image (EPI) sequence (ADNI-GO and ADNI-2: flip angle = 80^o^, spatial resolution = 3 × 3 × 3 mm^3^, slice thickness = 3.3 mm; ADNI-3: flip angle = 90^o^, slice thickness = 3.4 mm; see http://adni.loni.usc.edu/methods/documents/ for details) with TR/TE = 3,000/30 ms. Amyloid PET data were acquired from the approximately 50 to 70 minutes postinjection of florbetapir-fluorine-18 (^18^F-AV-45) (see the detailed description in http://adni.loni.usc.edu/data-samples/data-types/).

For the rsfMRI data, we applied the preprocessing procedures typical for rsfMRI signals using a 1000 Functional Connectomes Project script (version 1.1-beta; publicly available at https://www.nitrc.org/frs/?group_id=296) with some minor modifications [73]. Specifically, we performed motion correction, skull stripping, spatial smoothing (full width at half maximum (FWHM) = 4mm), temporal filtering (bandpass filter, approximately 0.01 to 0.1 Hz), and linear and quadratic temporal trends removal. The first 5 and last 5 rsfMRI volumes were discarded to allow for the magnetization to reach steady state and also to avoid the edge effect of the temporal filtering. We then co-registered each fMRI session to corresponding high-resolution anatomical images (T1-weighted MRI) and then to the 152-brain Montreal Neurological Institute (MNI-152) space. Our modifications of the original scripts include performing spatial registration between functional and anatomical images using align_epi_anat.py [74] in AFNI software [75] and resampling the preprocessed fMRI data into a spatial resolution of 3 × 3 × 3 mm^3^. We also skipped the nuisance regression of global signal, CSF signal, motion parameters to keep consistent with the previous study of the BOLD–CSF coupling [48]. The global brain signal and the CSF signal are the major interests of our study. The head motion, which had been suggested to cause rsfMRI changes [76], was recently found to be more likely a by-product associated with, rather than the cause of, the arousal-related global BOLD signal [44]. We thus skip the motion parameter regression to avoid attenuating the global BOLD signal. In a sensitivity analysis, we did assess the potential relationship between head motion and BOLD–CSF coupling as described below.

For the Amyloid PET data, we directly used SUVR data summarized in “UC Berkeley—AV45 Analysis [ADNI1,GO,2,3] (version: 07_28_19)” [69] (available at https://ida.loni.usc.edu/). Briefly, the florbetapir images were averaged, aligned spatially, and interpolated to a standard voxel size. The images were then spatially smoothed and then reregistered to the space of T1-weighted MRI in attempted to extract mean florbetapir uptake at gray matter. Notably, we used the composite region, including the whole cerebellum, brainstem/pons, and subcortical WM regions, as the reference region [50], and the cortical Aβ SUVR was calculated as the ratio of the mean florbetapir uptake at the gray matter and that at the composite reference region.

### Extraction of the global BOLD signal and the CSF signal

We extracted the mean BOLD signal from the gray matter regions of the cortex and regarded it as the global BOLD signal following the practice of previous studies by others [43,77]. A mask of the gray matter regions was defined based on the Harvard–Oxford cortical and subcortical structural atlases [78] (https://neurovault.org/collections/262/). We transformed the gray matter mask from the MNI-152 space back to the original space of each participant/session to avoid spatial blurring from the registration process with the same rationale to a previous study [48]. For each session, the fMRI signals were extracted from and averaged within the individual’s gray matter mask after all the preprocessing procedures but before the spatial registration to the MNI-152 space. The fMRI signals were also normalized to Z-score at each voxel before being averaged. Therefore, all the voxels have the equal fluctuation amplitude, and the amplitude of their average, i.e., the global BOLD signal, would reflect the level of global synchronization.

The CSF signals were extracted from the bottom slices of fMRI acquisition, which are near the bottom of the cerebellum for all the participants, to obtain high sensitivity to the CSF inflow effect as described previously [48]. The fMRI signal changes at the CSF regions are not due to BOLD effect since there are no blood. MR signals from spins undergoing repetitive radiofrequency (RF) pulses often take a few cycles to reach the steady state. Thus, fresh spins flowing into the imaging volume would result in an increase of fMRI signal intensity, which was called inflow effect [48,79]. It has been shown that this inflow effect size is much larger for CSF regions at the bottom of the imaging volume [48]. We thus obtained the CSF signals from the bottom slice similar to the previous study [48]. For each session, a CSF mask was manually defined at the bottom slice based on the T2*-weighted fMRI image, and its location was further confirmed with the T1-weighted structural image. The fMRI signals were extracted from and averaged within the individual’s CSF mask after several preprocessing procedures, including motion correction, skull stripping, and temporal filtering, but before the spatial registration to the MNI152 space.

### The coupling between the global BOLD signal and the CSF signal

We calculated the cross-correlation functions between the global BOLD signal and the CSF signal obtained through the above procedures to quantify their coupling, similarly to the previous study [48]. The cross-correlation functions show the Pearson correlations between the global BOLD signal and the CSF signal at different time lags. While the negative peak at the lag of +3 seconds is of a similar amplitude as the positive peak at the lag of −6 seconds in our study, it appeared to be stronger than the positive peak in the previous study with higher spatial and temporal resolutions [48]. We thus used the BOLD–CSF correlation at this negative peak (the lag of +3 seconds) for quantifying the strength of the BOLD–CSF coupling. Following the previous study [48], we also calculated the cross-correlation function between the negative (temporal) derivative of the global BOLD signal and the CSF signal. To test the statistical significance of the BOLD–CSF correlations, we used a permutation method to create a null distribution for the BOLD–CSF correlation. Specifically, we recalculated cross-correlation functions between the mismatched global BOLD signal and CSF signal after randomly permuting the session ID of the global BOLD signals. This process was repeated by 10,000 times to build the null distribution for the mean BOLD–CSF cross-correlation functions. The *p*-value for the BOLD–CSF correlations at different lags was obtained by comparing them against the null distribution.

### The relationship between the BOLD–CSF coupling and AD-related pathology

We quantified the BOLD–CSF coupling strength using their cross-correlation at the lag of +3 seconds (the negative peak of the cross-correlation function). We then related the BOLD–CSF coupling to age, gender, disease condition, and APOE gene. The association between the BOLD–CSF coupling and age was investigated across sessions (*N* = 158), and it was also compared across different subgroups, e.g., males versus females, using a linear mixed effect model. For the disease condition, we also tested whether the BOLD–CSF coupling strength (age and gender adjusted) changed with the severity of the AD-related symptoms (see details about the severity and staging of 4 conditions at http://adni.loni.usc.edu/study-design/). For APOE gene, we tested the relationship between the BOLD–CSF coupling strength (age and gender adjusted) and the number of APOE ε4 alleles. We repeated the same analysis for the augmented dataset with more AD patients and APOE ε4 alleles carriers.

To investigate whether the relationships of the BOLD–CSF coupling with age, gender, and APOE genotypes are driven by the differences between different disease groups, i.e., AD, MCI, SMC, and HC groups, we repeated the above analyses with controlling for the disease condition. To test whether the relationship between the BOLD–CSF coupling and age/gender/APOE ε4 allele number/disease condition was related to the cortical Aβ SUVR level, we repeated the above analyses by controlling for the cortical Aβ SUVR at baseline.

We also examined the associations between the BOLD–CSF coupling and AD-related markers, including the cortical Aβ level [80–82] and MMSE scores [83–85] at the baseline and their changes during the subsequent 2 years. The BOLD–CSF coupling strength was adjusted for age and gender effect before all subsequent analyses. The Aβ deposition at the gray matter regions was estimated with the whole-cortical Aβ SUVR, and we also estimated its 2-year change by subtracting its baseline value from that of the 2-year follow-up. The cortical Aβ SUVR was calculated with respect to 2 different reference regions, but its association with the BOLD–CSF coupling remained similar (Fig 3, S4 Fig). We also correlated the baseline MMSE score and its 2-year longitudinal change with the BOLD–CSF coupling. The above correlational analyses were also conducted across the participants by discarding some data. Specially, for the participants with data at more than 2 time points, we discarded those acquired at year 4 and later.

To exclude the possibility that the reported results are driven by the difference between the AD and HC groups in the BOLD–CSF coupling, we repeated the above correlational analyses by removing sessions from the AD and HC groups. Moreover, we also repeated the above analyses by using the BOLD–CSF correlations at different time lags to represent the BOLD–CSF coupling strength.

To examine the effect of head motion on our major results, we quantified the head motion using FD [86], which was widely used in brain imaging studies. The FD was computed as the sum of the absolute value of all 6 translational and rotational realignment parameters obtained at the motion correction reprocessing step [76]. We then used the mean FD averaged over all time points to represent the head motion level of each session [44]. We correlated the mean FD with age, baseline Aβ SUVR, the 2-year MMSE changes, and the BOLD–CSF coupling across all sessions. Furthermore, we retested the coupling–marker associations in Fig 3 with further controlling for the mean FD in addition to age and gender. We did not use motion-censoring approaches [76,87] to control and test the potential motion effects, since the BOLD–CSF coupling was estimated by their cross-correlation at the time lag of +3 seconds. The FD-based scrubbing procedures would concatenate time points that were not collected consecutively and thus affect the BOLD–CSF correlations at nonzero lags.

Although the majority (134 out of 158) of fMRI image sessions we used were collected by ADNI 2, there are data from ADNI 3 and ADNI GO. These different studies may use slightly different image parameters and/or experimental setups. To make sure that our major findings were not caused by any potential differences, we examined the associations between the BOLD–CSF coupling and AD pathologies using only the data from ADNI 2. We further included the study cohort (i.e., ADNI 2, 3, or GO) as a covariate and retested these associations.

### The role of the arousal state in the coupling–marker relationships

Since the arousal fluctuation is a major confounding factor in rsfMRI [26,88,89] and affects the BOLD–CSF coupling to a large extent [48], it would be appropriate to investigate the potential arousal effect on the relationships between the BOLD–CSF coupling and AD-related marker. We calculated the SD of the global BOLD signal for each session to quantify its fluctuation amplitude, which has been linked to the arousal state with a larger value suggesting a drowsy and sleepy state [37,48]. The global BOLD signal amplitude was adjusted for age and gender and then correlated with the BOLD–CSF coupling strength, the baseline amyloid PET, and the 2-year MMSE changes. Then, the associations between the BOLD–CSF coupling and the AD-related markers were reevaluated with further controlling for this global BOLD amplitude.

We also repeated the above analysis in Fig 4 using another fMRI-based arousal index [51] that is based on a spatial arousal template and thus utilizes the information independent from the global signal amplitude to estimate the arousal level. Specifically, we calculated the spatial correlation between single rsfMRI volumes and an arousal template [51] and then took the SD of this time course to quantify the arousal level of each rsfMRI sessions.

### Statistical analysis

Group comparisons of our sample characteristics (Table 1, S1 Table) were performed using 2-sample *t* test for continuous measures (i.e., age, MMSE and cortical Aβ SUVR) and Fisher exact test for categorical measures (i.e., gender and APOE ε4 allele negative/positive) across groups (AD, MCI, SMC, and HC). Significant differences (*p* < 0.05) for each comparison were marked with a bold format. The significance of the BOLD–CSF correlations was tested with a permutation method that shuffled the correspondence between the global BOLD signal and the CSF signal to build the null distributions for the BOLD–CSF correlation at different time lags, similar to the previous study [48]. Spearman correlation was used for quantifying the intersession and inter-subject associations between different quantities, some of which, e.g., the MMSE scores, showed a non-Gaussian distribution [90]. Because some sessions were collected from the same participant, we employed a linear mixed effect model to assess the statistical significance of various relationships in all session-based analyses. Specifically, subject-specific random intercepts were included to account for the dependency of intra-subject sessions. It was worth noting that the linear mixed effect model has been used even when we estimated and adjusted the effect of age, gender, and all other confounding factors. The linear mixed model was implemented by the “lme4” package of R [91]. The corresponding *p*-values were computed using the R package “lmerTest” based on the Satterthwaite degrees of freedom method [92]. We also validated the major findings in a subject-based analysis based on a simple linear regression model. In this study, a *p*-value less than 0.05 was regarded as statistical significance.

## Supporting information

S1 Fig The dependency of the BOLD–CSF coupling on AD risk factors and disease conditions in the augmented dataset. (**A**) When we increased the sample size of AD and HC session (*N* = 199), the BOLD–CSF coupling shows a significant correlation with age across sessions (Spearman’s *r* = 0.20, *p* = 0.02). AD, MCI, SMC, and HC sessions are colored with blue, light gray, dark gray, and orange, respectively. Each dot represents a session. (**B**) Male participants have larger amplitudes of this BOLD–CSF coupling compared with female ones (*p* = 0.038) in this augmented dataset. (**C**) The strength of the BOLD–CSF coupling, after adjusting the age and gender effects, also decreases gradually (*p* = 0.005) along with the increased severity of disease condition, i.e., the axis of HC–SMC–MCI–AD. Importantly, significant differences can be found not only between the HC and MCI groups (*p* = 0.045) but also between the HC and AD sessions (*p* = 0.0078). (**D**) The age- and gender-adjusted BOLD–CSF coupling is also significantly correlated with the APOE ε4 allele number (*N* = 184, *p* = 0.041) across augmented sessions. Error bar in this figure represents the SEM. The underlying data can be found in S1 Data. AD, Alzheimer disease; APOE, apolipoprotein E; BOLD, blood oxygen level–dependent; CSF, cerebrospinal fluid; HC, healthy control; MCI, mild cognitive impairment; SEM, standard error of the mean; SMC, significant memory concern.
(TIF)

S2 Fig The dependency of the BOLD–CSF coupling on AD risk factors with controlling for disease condition. (**A**) The strength of the BOLD–CSF coupling adjusted for the disease condition (i.e., the effects of AD, MCI, SMC, and HC groups) shows a significant correlation (Spearman’s *r* = 0.22, *p* = 0.020) with age across the 158 sessions. (**B**) Male participants showed a larger amplitude of the BOLD–CSF coupling as compared with females (*p* = 0.032) after controlling for disease condition. (**C**) The age-, gender-, and disease condition- adjusted BOLD–CSF coupling amplitude gradually decreased as the APOE ε4 allele number increases, but this change is not statistically significant (*p* = 0.22). Error bar in this figure represents the SEM. The underlying data can be found in S1 Data. AD, Alzheimer disease; APOE, apolipoprotein E; BOLD, blood oxygen level–dependent; HC, healthy control; CSF, cerebrospinal fluid; MCI, mild cognitive impairment; SEM, standard error of the mean; SMC, significant memory concern.
(TIF)

S3 Fig The relationship between AD markers and the BOLD–CSF coupling with excluding the HC and AD data. (**A**, **B**) The BOLD–CSF coupling adjusted for age and gender is significantly correlated (Spearman’s *r* = 0.20, *p* = 0.026) with the gray matter Aβ SUVRs (**A**) but not the 2-year longitudinal Aβ changes (**B**) across sessions of the MCI and SMC groups. (**C**, **D**) The BOLD–CSF coupling adjusted for age and gender is significantly correlated with (Spearman’s *r* = −0.24, *p* = 0.016) the 2-year longitudinal changes of MMSE scores (**D**) but not the baseline values (**C**) across sessions from MCI and SMC. Each dot represents a session. MCI and SMC sessions are colored with light gray and dark gray, respectively. The underlying data can be found in S1 Data. Aβ, amyloid-β; AD, Alzheimer disease; BOLD, blood oxygen level–dependent; CSF, cerebrospinal fluid; HC, healthy control; MCI, mild cognitive impairment; MMSE, Mini-Mental State Examination; SMC, significant memory concern; SUVR, standardized uptake value ratio.
(TIF)

S4 Fig The association between the BOLD–CSF coupling and Aβ-PET SUVR normalized with eroded white matter. (**A**, **B**) The BOLD–CSF coupling adjusted for age and gender is significantly correlated (Spearman’s *r* = 0.19, *p* = 0.016) with the gray matter SUVRs (**A**) but not the 2-year longitudinal SUVR changes (**B**) when the reference region is defined as the eroded white matter [50]. The results here with the alternative reference region are very similar to the results in **Fig 3A** and **3B**. Each dot represents a session. AD, MCI, SMC, and HC sessions are colored with blue, light gray, dark gray, and orange, respectively. The underlying data can be found in S1 Data. Aβ, amyloid-β; AD, Alzheimer disease; BOLD, blood oxygen level–dependent; CSF, cerebrospinal fluid; HC, healthy control; MCI, mild cognitive impairment; SMC, significant memory concern; SUVR, standardized uptake value ratio.
(TIF)

S5 Fig The relationship between AD markers and BOLD–CSF coupling across participants. (**A**, **B**) BOLD–CSF coupling adjusted for age and gender is significantly correlated (Spearman’s *r* = 0.21, *p* = 0.022, *N* = 118) with the gray matter Aβ SUVRs (**A**) but not the 2-year longitudinal Aβ changes (**B**) across participants. The composite reference region is the same as **Fig 3A** and **3B**. (**C**, **D**) The association between the age- and gender-adjusted BOLD–CSF coupling and the 2-year longitudinal MMSE changes is significant (Spearman’s *r* = −0.21, *p* = 0.026) (**D**) but its correlation with the baseline MMSE score is not significant (*p* = 0.99) (**C**). The linear regression lines were estimated based on the linear least-squares fitting [49]. Each dot represents a participant. AD, MCI, SMC, and HC participants are colored with blue, light gray, dark gray, and orange, respectively. The underlying data can be found in S1 Data. Aβ, amyloid-β; AD, Alzheimer disease; BOLD, blood oxygen level–dependent; CSF, cerebrospinal fluid; HC, healthy control; MCI, mild cognitive impairment; MMSE, Mini-Mental State Examination; SMC, significant memory concern; SUVR, standardized uptake value ratio.
(TIF)

S6 Fig The relationship between AD markers and the motion-adjusted BOLD–CSF coupling across sessions. (**A**, **B**) The BOLD–CSF coupling adjusted for age, gender, and mean FD is significantly correlated (Spearman’s *r* = 0.20, *p* = 0.023, *N* = 158, the linear mixed model with Satterthwaite method) with the cortical Aβ SUVRs at baseline (**A**) but not their changes in the following 2 years (**B**). (**C**, **D**) The association between the age-, gender-, and mean FD-adjusted BOLD–CSF coupling and the 2-year longitudinal MMSE changes is significant (Spearman’s *r* = −0.20, *p* = 0.012) (**D**) but its correlation with the baseline MMSE score is not significant (*p* = 0.85) (**C**). The linear regression lines were estimated based on the linear least-squares fitting. Each dot represents a single session. The underlying data can be found in S1 Data. Aβ, amyloid-β; AD, Alzheimer disease; BOLD, blood oxygen level–dependent; CSF, cerebrospinal fluid; MMSE, Mini-Mental State Examination; SUVR, standardized uptake value ratio.
(TIF)

S7 Fig The head motion, quantified with mean FD, was not correlated with the AD-related markers. The mean FD was not significantly correlated with age, cortical Aβ SUVRs at baseline, 2-year MMSE change, or the BOLD–CSF coupling across sessions (*p* > 0.05, linear mix model adjustment was used). The linear regression lines were estimated based on the linear least-squares fitting. AD, MCI, SMC, and HC sessions are colored with blue, light gray, dark gray, and orange, respectively. The underlying data can be found in S1 Data. Aβ, amyloid-β; AD, Alzheimer disease; BOLD, blood oxygen level–dependent; CSF, cerebrospinal fluid; FD, framewise displacement; HC, healthy control; MCI, mild cognitive impairment; MMSE, Mini-Mental State Examination; SMC, significant memory concern; SUVR, standardized uptake value ratio.
(TIF)

S8 Fig The relationship between AD markers and BOLD–CSF coupling across sessions in ADNI 2 or when controlling the cohort variables. (**A**–**D**) Similar to the main finding in **Fig 3**, the BOLD–CSF coupling is significantly correlated with cortical Aβ (**A**) and MMSE changes in the subsequent 2 years (**D**) across sessions in ADNI 2. (**E**–**H**) Very similar results (to **Fig 3**) were found when we regressed the cohort information (categorical variables) and repeated the coupling–marker correlation analyses. The underlying data can be found in S1 Data. Aβ, amyloid-β; AD, Alzheimer disease; ADNI, Alzheimer’s Disease Neuroimaging Initiative; BOLD, blood oxygen level–dependent; CSF, cerebrospinal fluid; MMSE, Mini-Mental State Examination.
(TIF)

S9 Fig Relationships between AD-related markers and the BOLD–CSF correlations at different time lags. (**A**) The cortical Aβ SUVRs and (**B**) the 2-year longitudinal MMS changes were correlated (Spearman’s *r*, across 158 sessions) with the BOLD–CSF correlations at different time lags after adjusting for age and gender. Black dots indicate significant correlations (*p* < 0.05). Red arrows indicate the lag (+3 seconds) that we used in the “BOLD–CSF coupling” (as **Figs 2–4**). The underlying data can be found in S1 Data. Aβ, amyloid-β; AD, Alzheimer disease; BOLD, blood oxygen level–dependent; CSF, cerebrospinal fluid; SUVR, standardized uptake value ratio.
(TIF)

S10 Fig The dependency of the BOLD–CSF coupling on AD risk factors and disease conditions with controlling for cortical Aβ SUVR at baseline. (**A**) The BOLD–CSF coupling adjusted for the cortical Aβ SUVR shows a significant correlation (Spearman’s *r* = 0.19, *p* = 0.049) with age across the 158 sessions. (**B**) Male participants showed a stronger BOLD–CSF coupling (adjusted for cortical Aβ SUVR) as compared with females (*p* = 0.06). (**C**) The BOLD–CSF coupling, after adjusting age, gender, and cortical Aβ SUVR, gradually decrease from the HC, to SMC, to MCI, and then to AD group, but the change is not statistically significant (*p* = 0.32). (**D**) The age-, gender-, and cortical-Aβ SUVR adjusted BOLD–CSF coupling is not significantly (*p* = 0.86) correlated with the APOE ε4 allele. Error bar in this figure represents the SEM. The underlying data can be found in S1 Data. Aβ, amyloid-β; AD, Alzheimer disease; APOE, apolipoprotein E; BOLD, blood oxygen level–dependent; CSF, cerebrospinal fluid; HC, healthy control; MCI, mild cognitive impairment; SEM, standard error of the mean; SMC, significant memory concern; SUVR, standardized uptake value ratio.
(TIF)

S11 Fig The relationships between the BOLD–CSF coupling and AD-related markers are not affected by the inter-subject variability in arousal. (**A**) The association between the BOLD–CSF coupling (adjusted for age and gender) and the arousal index has a similar trend as **Fig 4A**. (**B**, **C**) The arousal index, adjusted for age and gender, is not significantly correlated with either the cortical Aβ level (**B**) or the 2-year longitudinal change of MMSE score (**C**). (**D**, **E**) The BOLD–CSF coupling remains to be significantly correlated with the cortical Aβ level (**D**) and the 2-year MMSE changes (**E**) after adjusting for age, gender, and arousal index. AD, MCI, SMC, and HC sessions are colored with blue, light gray, dark gray, and orange, respectively. Each dot represents a session. The underlying data can be found in S1 Data. Aβ, amyloid-β; AD, Alzheimer disease; BOLD, blood oxygen level–dependent; CSF, cerebrospinal fluid; HC, healthy control; MCI, mild cognitive impairment; MMSE, Mini-Mental State Examination; SMC, significant memory concern.
(TIF)

S12 Fig The global BOLD signal in percentage changes and cross-session variation of the BOLD–CSF cross-correlation function. (**A**) The global BOLD signal and the CSF signal (both were represented with the percentage changes) from a representative participant showed corresponding changes (indicated by black arrows). (**B**) The BOLD–CSF cross-correlation functions from each of 158 sessions (gray curves). The black line represents the mean, whereas the error bars represent the SD across all the session. The underlying data can be found in S1 Data. BOLD, blood oxygen level–dependent; CSF, cerebrospinal fluid; SD, standard deviation.
(TIF)

S1 Table Participant baseline characteristics of the augmented sample. *p*-Values are derived from 2-sample *t* test for continuous measures and from Fisher exact test for categorical measures. The underlying data can be found in S1 Data. 24 m follow-up, 24 months follow-up; Aβ florbetapir SUVR, the whole cortical amyloid beta from PET AV45 analysis normalized composite reference region; AD, Alzheimer disease participants; APOE ε4 status (Neg/Pos), not APOE ε4 carrier/APOE ε4 carrier; HC, healthy control; M/F, male/female; M/SD, mean/standard deviation; MCI, mild cognition impairment; MMSE, Mini-Mental State Examination; SMC, significant memory concern.
(DOCX)

S1 Data The data and code for reproducing all main and Supporting information figures. This zipped folder contains Excel tables organized by figure and table, (i.e., Fig 1B and 1C; Fig 2A–2D; Fig 3A–3D; Fig 4A–4E; S1A–S1D Fig; S2A–S2C Fig; S3A–S3D Fig; S4A and S4B Fig; S5A–S5D Fig; S6A–S6D Fig; S7A–S7D Fig; S8A–S8H Fig; S9A and S9B Fig; S10A–S10D Fig; S11A–S11E Fig; S12A and S12B Fig; Table 1; S1 Table) within which the data for individual panels are displayed in separate tabs/sheets, as well as the scripts used to generate the all above figures and tables in this manuscript.
(ZIP)

## Abbreviations

Aβ: amyloid-β
AD: Alzheimer disease
ADNI: Alzheimer’s Disease Neuroimaging Initiative
APOE: apolipoprotein E
AQP4: aquaporin-4
BOLD: blood oxygen level–dependent
CSF: cerebrospinal fluid
EEG: electroencephalography
EPI: echo-planar image
FD: framewise displacement
HC: healthy control
ICA: internal carotid artery
IRB: institutional review board
ISF: interstitial fluid
MCI: mild cognitive impairment
MMSE: Mini-Mental State Examination
MNI: Montreal Neurological Institute
PET: positron emission tomography
RF: radiofrequency
rsfMRI: resting-state functional magnetic resonance imaging
SD: standard deviation
SMC: significant memory concern
SST: sequential spectral transition
SUVR: standardized uptake value ratio
