# Supplementary figures and images for: Reduced coupling between cerebrospinal fluid flow and global brain activity is linked to Alzheimer disease–related pathology
Source: PLoS Biol. 2021 Jun 1;19(6):e3001233. doi: 10.1371/journal.pbio.3001233 (PMC8168893; doi:10.1371/journal.pbio.3001233)

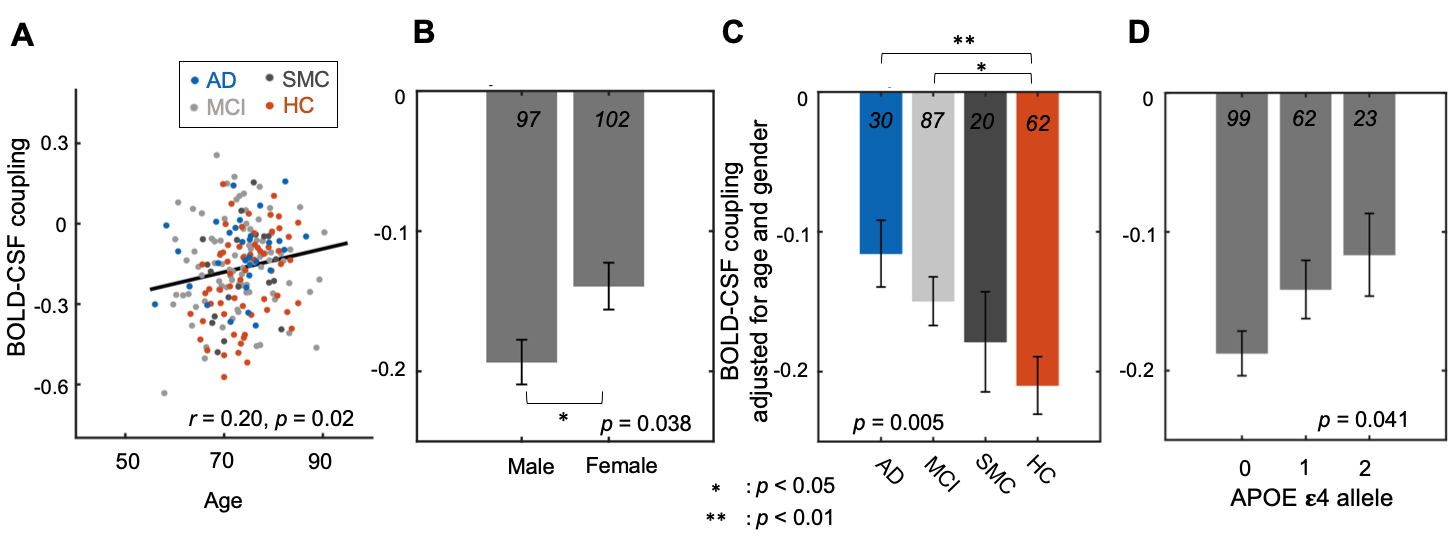

Supplement: S1 Fig — (A) When we increased the sample size of AD and HC session (N = 199), the BOLD–CSF coupling shows a significant correlation with age across sessions (Spearman’s r = 0.20, p = 0.02). AD, MCI, SMC, and HC sessions are colored with blue, light gray, dark gray, and orange, respectively. Each dot represents a session. (B) Male participants have larger amplitudes of this BOLD–CSF coupling compared with female ones (p = 0.038) in this augmented dataset. (C) The strength of the BOLD–CSF coupling, after adjusting the age and gender effects, also decreases gradually (p = 0.005) along with the increased severity of disease condition, i.e., the axis of HC–SMC–MCI–AD. Importantly, significant differences can be found not only between the HC and MCI groups (p = 0.045) but also between the HC and AD sessions (p = 0.0078). (D) The age- and gender-adjusted BOLD–CSF coupling is also significantly correlated with the APOE ε4 allele number (N = 184, p = 0.041) across augmented sessions. Error bar in this figure represents the SEM. The underlying data can be found in S1 Data. AD, Alzheimer disease; APOE, apolipoprotein E; BOLD, blood oxygen level–dependent; CSF, cerebrospinal fluid; HC, healthy control; MCI, mild cognitive impairment; SEM, standard error of the mean; SMC, significant memory concern. (TIF) [file pbio.3001233.s001.tif]

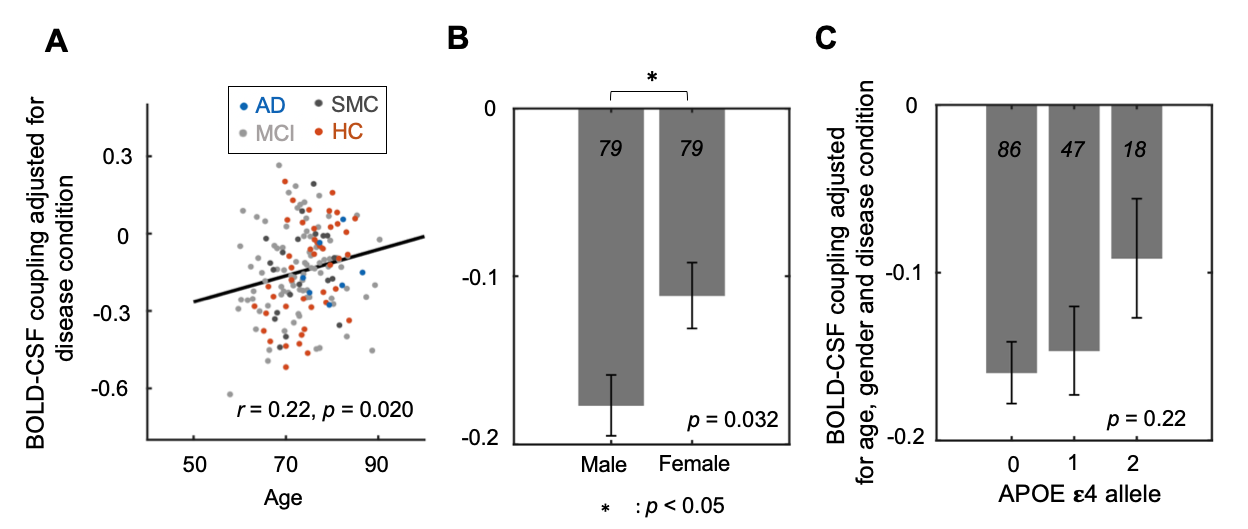

Supplement: S2 Fig — (A) The strength of the BOLD–CSF coupling adjusted for the disease condition (i.e., the effects of AD, MCI, SMC, and HC groups) shows a significant correlation (Spearman’s r = 0.22, p = 0.020) with age across the 158 sessions. (B) Male participants showed a larger amplitude of the BOLD–CSF coupling as compared with females (p = 0.032) after controlling for disease condition. (C) The age-, gender-, and disease condition- adjusted BOLD–CSF coupling amplitude gradually decreased as the APOE ε4 allele number increases, but this change is not statistically significant (p = 0.22). Error bar in this figure represents the SEM. The underlying data can be found in S1 Data. AD, Alzheimer disease; APOE, apolipoprotein E; BOLD, blood oxygen level–dependent; HC, healthy control; CSF, cerebrospinal fluid; MCI, mild cognitive impairment; SEM, standard error of the mean; SMC, significant memory concern. (TIF) [file pbio.3001233.s002.tif]

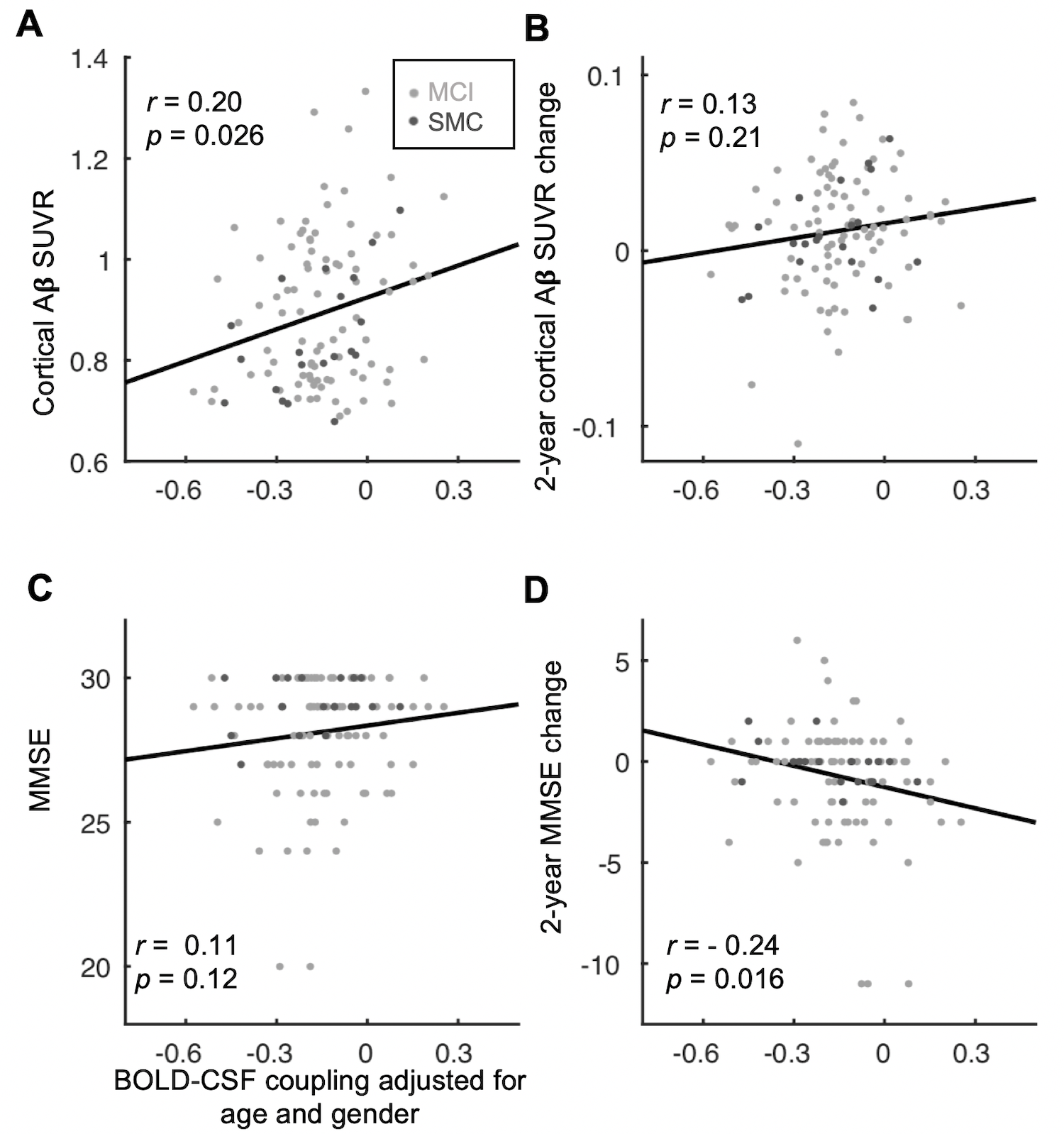

Supplement: S3 Fig — (A, B) The BOLD–CSF coupling adjusted for age and gender is significantly correlated (Spearman’s r = 0.20, p = 0.026) with the gray matter Aβ SUVRs (A) but not the 2-year longitudinal Aβ changes (B) across sessions of the MCI and SMC groups. (C, D) The BOLD–CSF coupling adjusted for age and gender is significantly correlated with (Spearman’s r = −0.24, p = 0.016) the 2-year longitudinal changes of MMSE scores (D) but not the baseline values (C) across sessions from MCI and SMC. Each dot represents a session. MCI and SMC sessions are colored with light gray and dark gray, respectively. The underlying data can be found in S1 Data. Aβ, amyloid-β; AD, Alzheimer disease; BOLD, blood oxygen level–dependent; CSF, cerebrospinal fluid; HC, healthy control; MCI, mild cognitive impairment; MMSE, Mini-Mental State Examination; SMC, significant memory concern; SUVR, standardized uptake value ratio. (TIF) [file pbio.3001233.s003.tif]

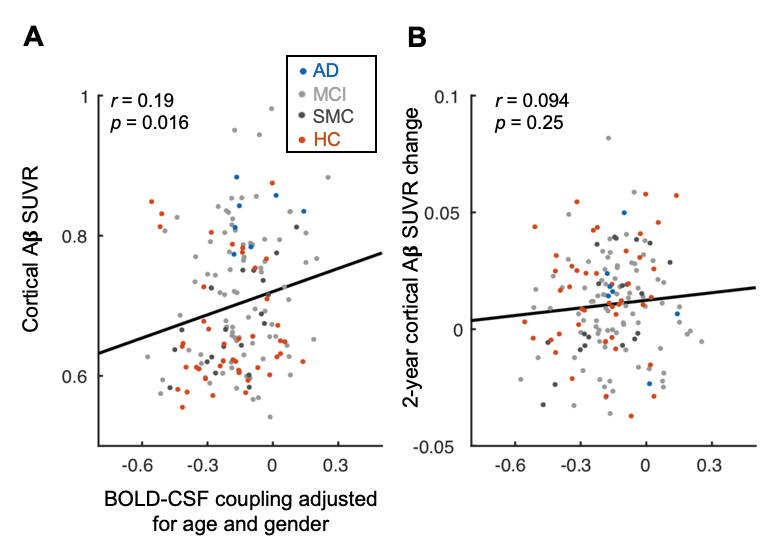

Supplement: S4 Fig — (A, B) The BOLD–CSF coupling adjusted for age and gender is significantly correlated (Spearman’s r = 0.19, p = 0.016) with the gray matter SUVRs (A) but not the 2-year longitudinal SUVR changes (B) when the reference region is defined as the eroded white matter [50]. The results here with the alternative reference region are very similar to the results in Fig 3A and 3B. Each dot represents a session. AD, MCI, SMC, and HC sessions are colored with blue, light gray, dark gray, and orange, respectively. The underlying data can be found in S1 Data. Aβ, amyloid-β; AD, Alzheimer disease; BOLD, blood oxygen level–dependent; CSF, cerebrospinal fluid; HC, healthy control; MCI, mild cognitive impairment; SMC, significant memory concern; SUVR, standardized uptake value ratio. (TIF) [file pbio.3001233.s004.tif]

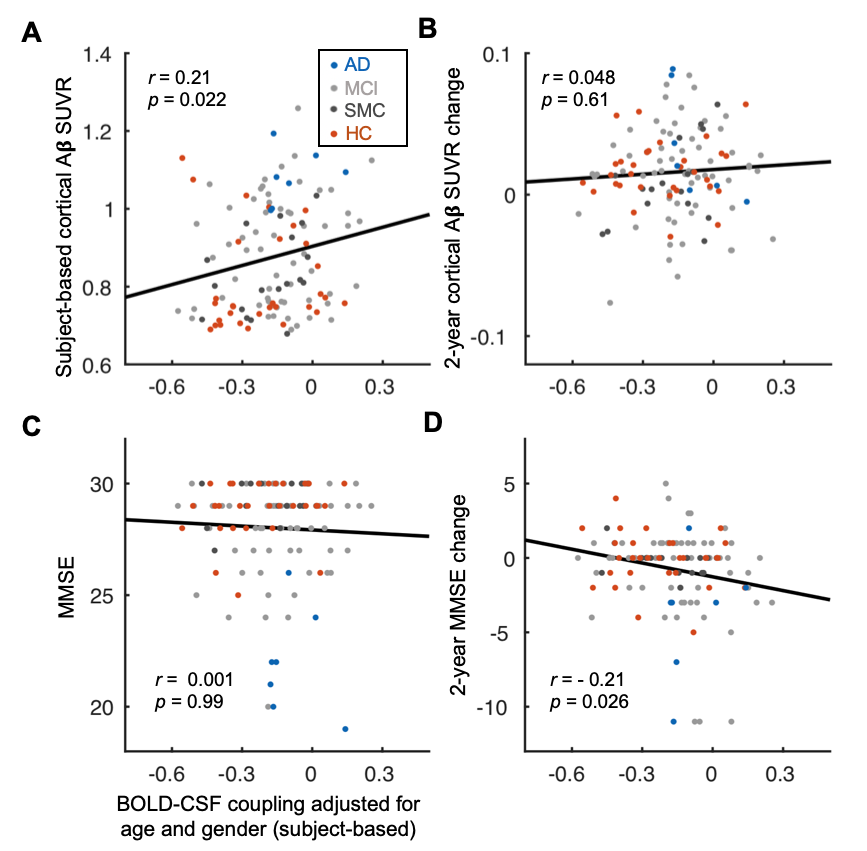

Supplement: S5 Fig — (A, B) BOLD–CSF coupling adjusted for age and gender is significantly correlated (Spearman’s r = 0.21, p = 0.022, N = 118) with the gray matter Aβ SUVRs (A) but not the 2-year longitudinal Aβ changes (B) across participants. The composite reference region is the same as Fig 3A and 3B. (C, D) The association between the age- and gender-adjusted BOLD–CSF coupling and the 2-year longitudinal MMSE changes is significant (Spearman’s r = −0.21, p = 0.026) (D) but its correlation with the baseline MMSE score is not significant (p = 0.99) (C). The linear regression lines were estimated based on the linear least-squares fitting [49]. Each dot represents a participant. AD, MCI, SMC, and HC participants are colored with blue, light gray, dark gray, and orange, respectively. The underlying data can be found in S1 Data. Aβ, amyloid-β; AD, Alzheimer disease; BOLD, blood oxygen level–dependent; CSF, cerebrospinal fluid; HC, healthy control; MCI, mild cognitive impairment; MMSE, Mini-Mental State Examination; SMC, significant memory concern; SUVR, standardized uptake value ratio. (TIF) [file pbio.3001233.s005.tif]

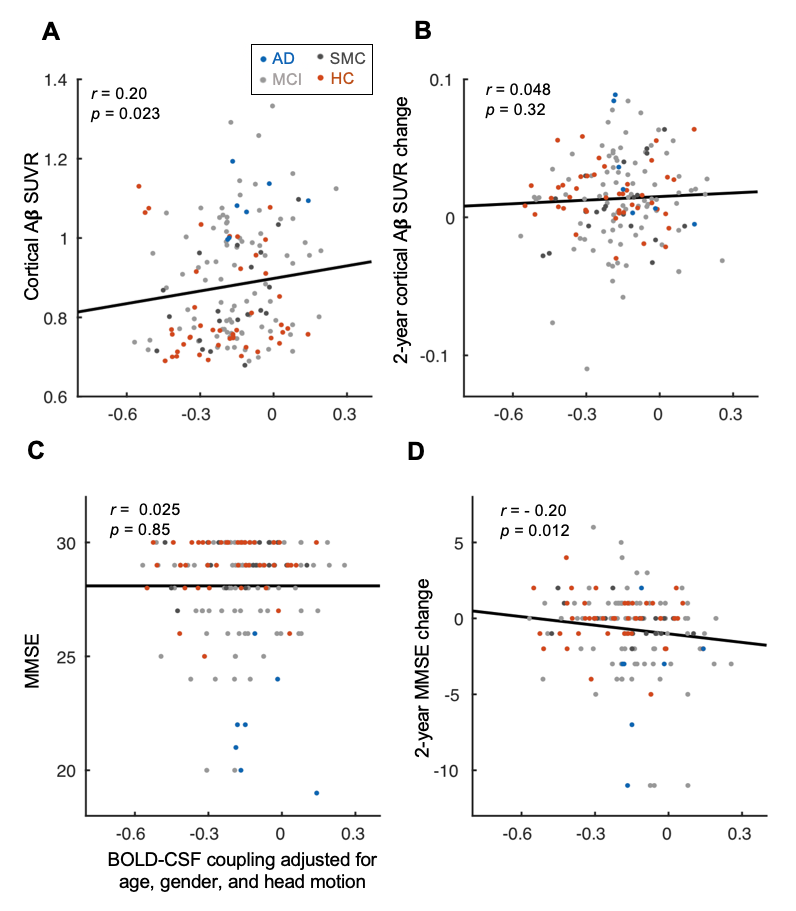

Supplement: S6 Fig — (A, B) The BOLD–CSF coupling adjusted for age, gender, and mean FD is significantly correlated (Spearman’s r = 0.20, p = 0.023, N = 158, the linear mixed model with Satterthwaite method) with the cortical Aβ SUVRs at baseline (A) but not their changes in the following 2 years (B). (C, D) The association between the age-, gender-, and mean FD-adjusted BOLD–CSF coupling and the 2-year longitudinal MMSE changes is significant (Spearman’s r = −0.20, p = 0.012) (D) but its correlation with the baseline MMSE score is not significant (p = 0.85) (C). The linear regression lines were estimated based on the linear least-squares fitting. Each dot represents a single session. The underlying data can be found in S1 Data. Aβ, amyloid-β; AD, Alzheimer disease; BOLD, blood oxygen level–dependent; CSF, cerebrospinal fluid; MMSE, Mini-Mental State Examination; SUVR, standardized uptake value ratio. (TIF) [file pbio.3001233.s006.tif]

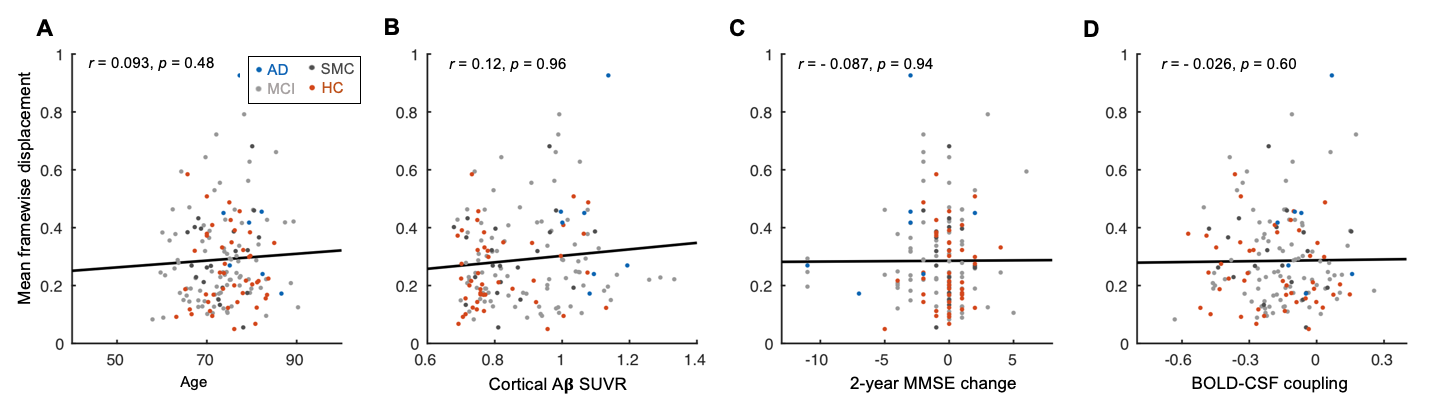

Supplement: S7 Fig — The mean FD was not significantly correlated with age, cortical Aβ SUVRs at baseline, 2-year MMSE change, or the BOLD–CSF coupling across sessions (p > 0.05, linear mix model adjustment was used). The linear regression lines were estimated based on the linear least-squares fitting. AD, MCI, SMC, and HC sessions are colored with blue, light gray, dark gray, and orange, respectively. The underlying data can be found in S1 Data. Aβ, amyloid-β; AD, Alzheimer disease; BOLD, blood oxygen level–dependent; CSF, cerebrospinal fluid; FD, framewise displacement; HC, healthy control; MCI, mild cognitive impairment; MMSE, Mini-Mental State Examination; SMC, significant memory concern; SUVR, standardized uptake value ratio. (TIF) [file pbio.3001233.s007.tif]

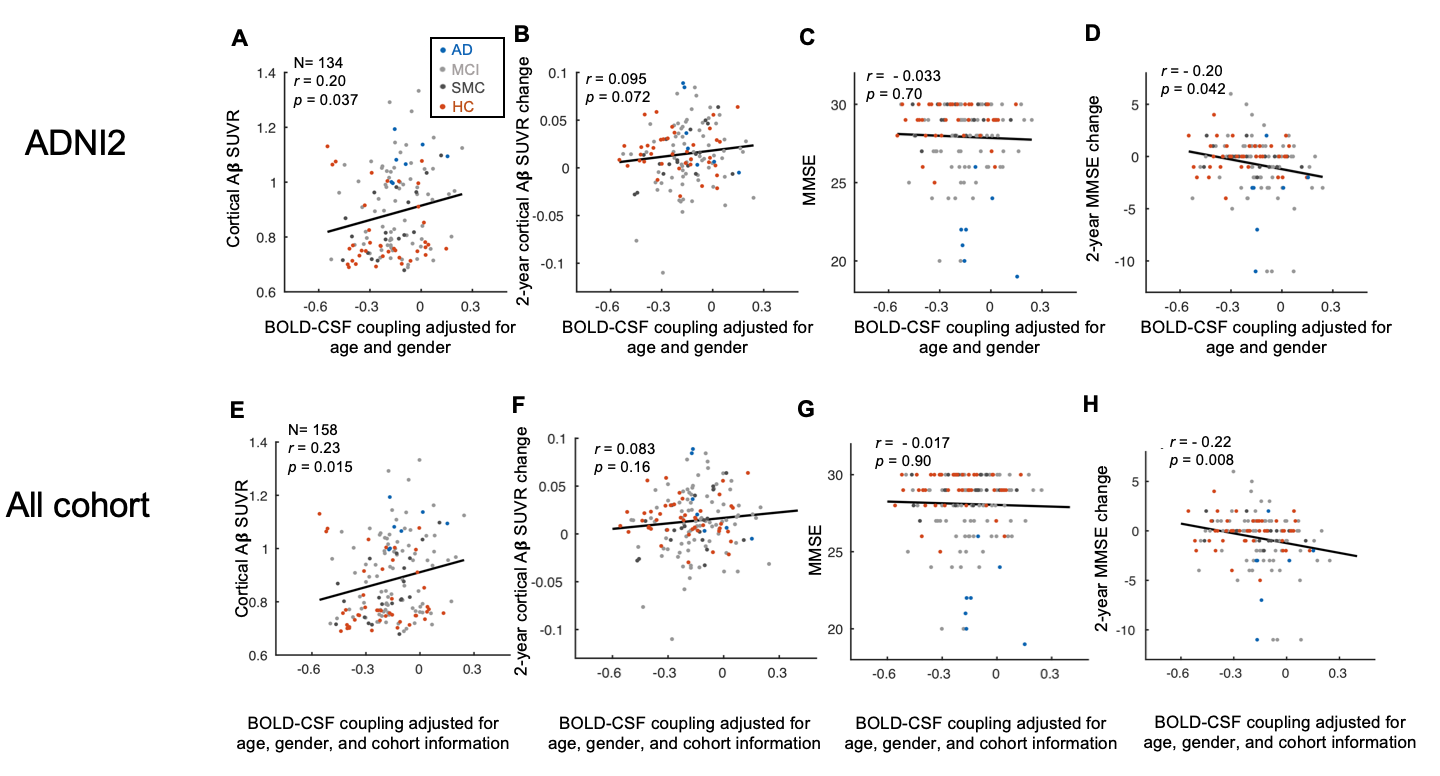

Supplement: S8 Fig — (A–D) Similar to the main finding in Fig 3, the BOLD–CSF coupling is significantly correlated with cortical Aβ (A) and MMSE changes in the subsequent 2 years (D) across sessions in ADNI 2. (E–H) Very similar results (to Fig 3) were found when we regressed the cohort information (categorical variables) and repeated the coupling–marker correlation analyses. The underlying data can be found in S1 Data. Aβ, amyloid-β; AD, Alzheimer disease; ADNI, Alzheimer’s Disease Neuroimaging Initiative; BOLD, blood oxygen level–dependent; CSF, cerebrospinal fluid; MMSE, Mini-Mental State Examination. (TIF) [file pbio.3001233.s008.tif]

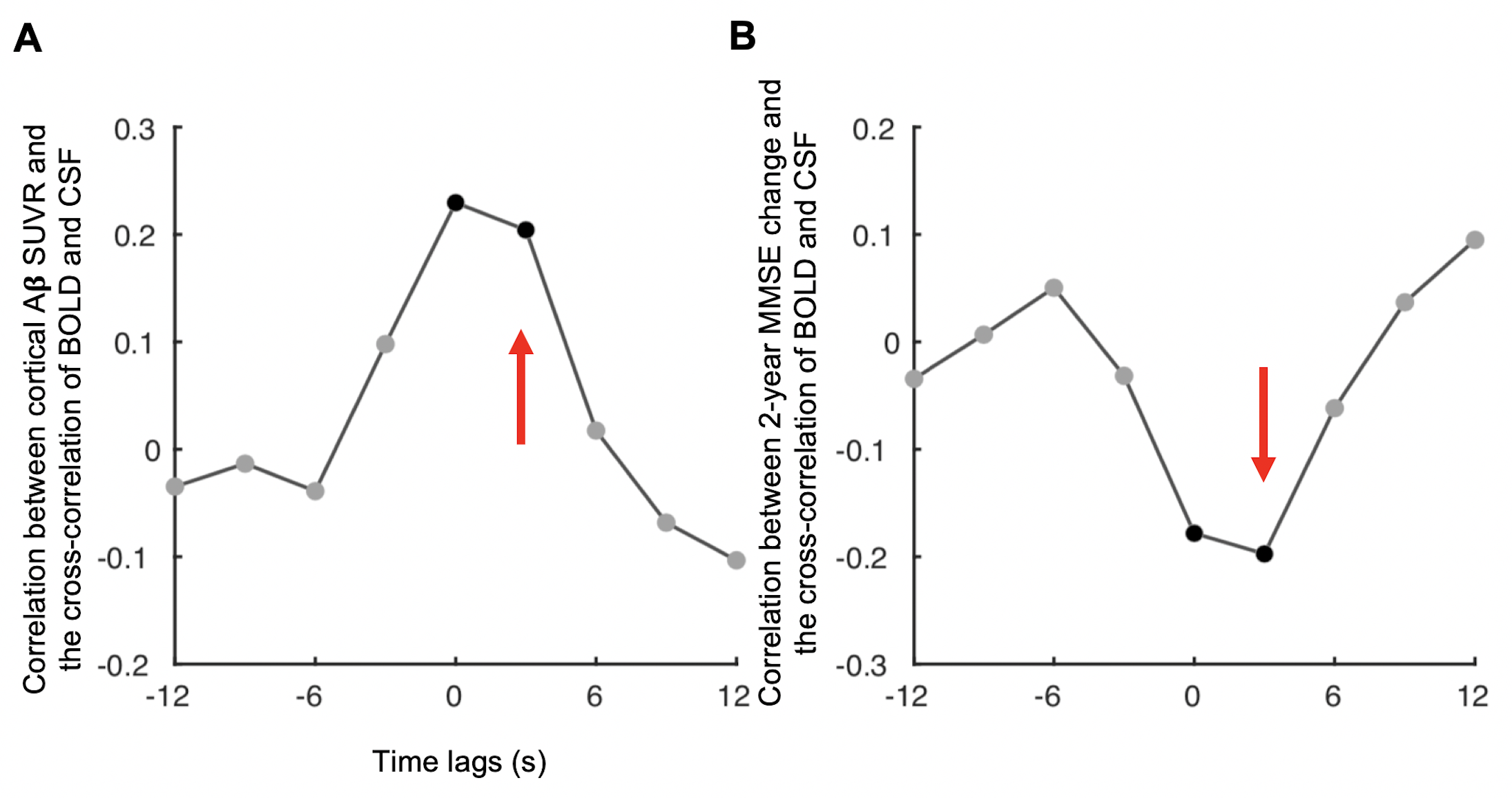

Supplement: S9 Fig — (A) The cortical Aβ SUVRs and (B) the 2-year longitudinal MMS changes were correlated (Spearman’s r, across 158 sessions) with the BOLD–CSF correlations at different time lags after adjusting for age and gender. Black dots indicate significant correlations (p < 0.05). Red arrows indicate the lag (+3 seconds) that we used in the “BOLD–CSF coupling” (as Figs 2–4). The underlying data can be found in S1 Data. Aβ, amyloid-β; AD, Alzheimer disease; BOLD, blood oxygen level–dependent; CSF, cerebrospinal fluid; SUVR, standardized uptake value ratio. (TIF) [file pbio.3001233.s009.tif]

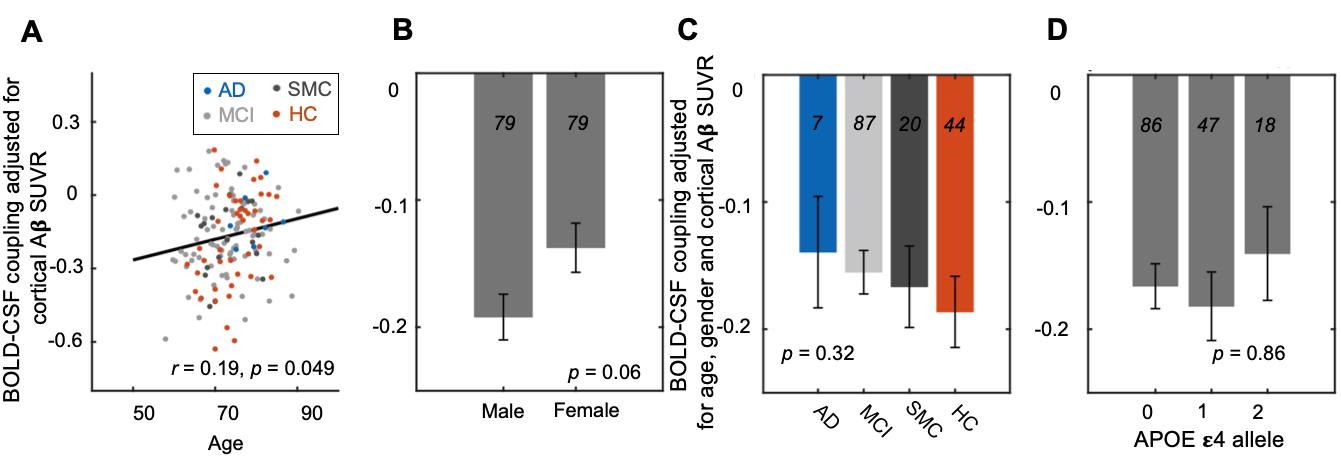

Supplement: S10 Fig — (A) The BOLD–CSF coupling adjusted for the cortical Aβ SUVR shows a significant correlation (Spearman’s r = 0.19, p = 0.049) with age across the 158 sessions. (B) Male participants showed a stronger BOLD–CSF coupling (adjusted for cortical Aβ SUVR) as compared with females (p = 0.06). (C) The BOLD–CSF coupling, after adjusting age, gender, and cortical Aβ SUVR, gradually decrease from the HC, to SMC, to MCI, and then to AD group, but the change is not statistically significant (p = 0.32). (D) The age-, gender-, and cortical-Aβ SUVR adjusted BOLD–CSF coupling is not significantly (p = 0.86) correlated with the APOE ε4 allele. Error bar in this figure represents the SEM. The underlying data can be found in S1 Data. Aβ, amyloid-β; AD, Alzheimer disease; APOE, apolipoprotein E; BOLD, blood oxygen level–dependent; CSF, cerebrospinal fluid; HC, healthy control; MCI, mild cognitive impairment; SEM, standard error of the mean; SMC, significant memory concern; SUVR, standardized uptake value ratio. (TIF) [file pbio.3001233.s010.tif]

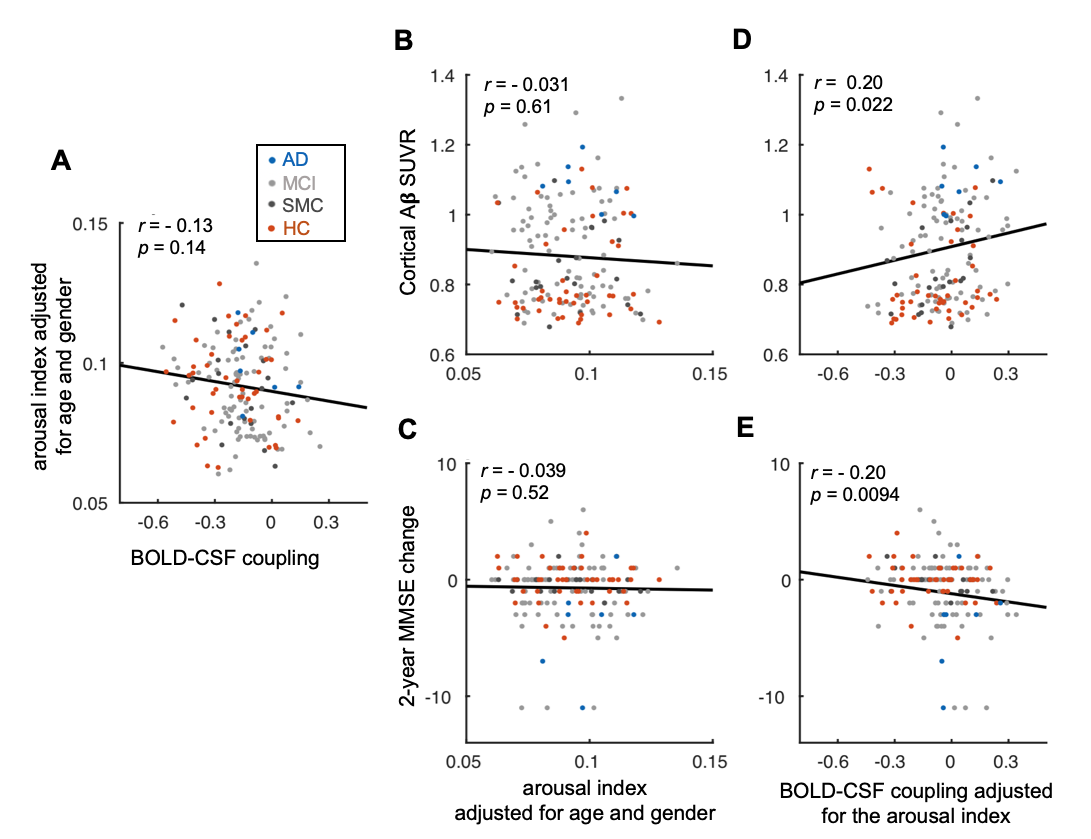

Supplement: S11 Fig — (A) The association between the BOLD–CSF coupling (adjusted for age and gender) and the arousal index has a similar trend as Fig 4A. (B, C) The arousal index, adjusted for age and gender, is not significantly correlated with either the cortical Aβ level (B) or the 2-year longitudinal change of MMSE score (C). (D, E) The BOLD–CSF coupling remains to be significantly correlated with the cortical Aβ level (D) and the 2-year MMSE changes (E) after adjusting for age, gender, and arousal index. AD, MCI, SMC, and HC sessions are colored with blue, light gray, dark gray, and orange, respectively. Each dot represents a session. The underlying data can be found in S1 Data. Aβ, amyloid-β; AD, Alzheimer disease; BOLD, blood oxygen level–dependent; CSF, cerebrospinal fluid; HC, healthy control; MCI, mild cognitive impairment; MMSE, Mini-Mental State Examination; SMC, significant memory concern. (TIF) [file pbio.3001233.s011.tif]

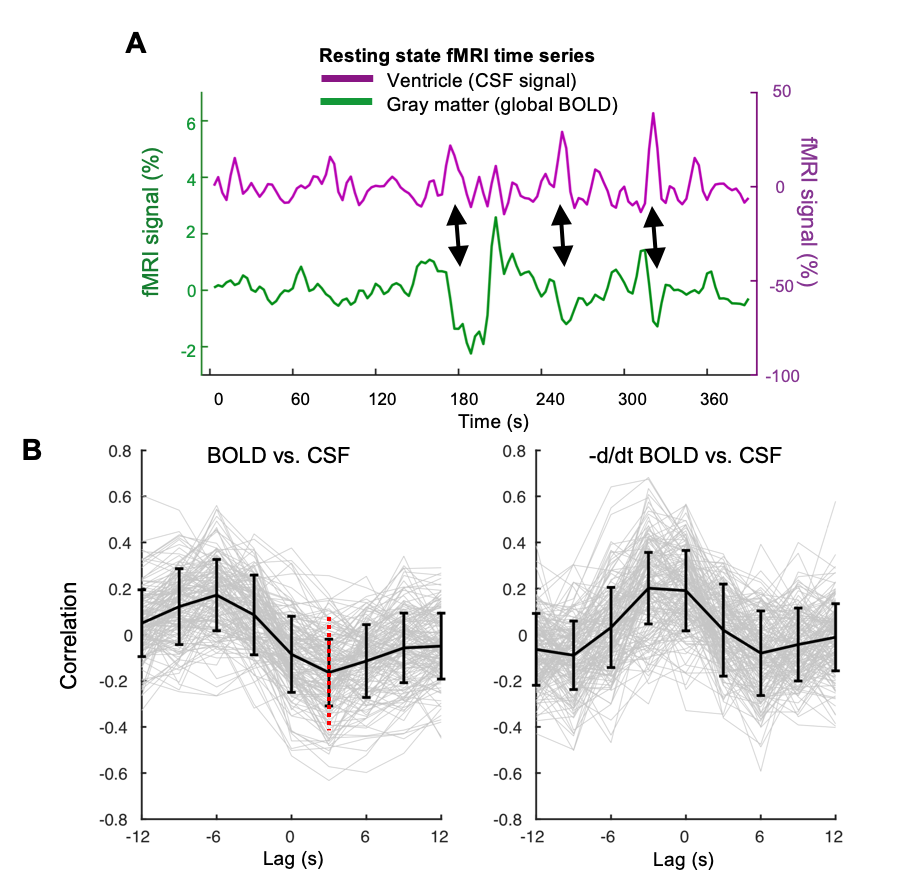

Supplement: S12 Fig — (A) The global BOLD signal and the CSF signal (both were represented with the percentage changes) from a representative participant showed corresponding changes (indicated by black arrows). (B) The BOLD–CSF cross-correlation functions from each of 158 sessions (gray curves). The black line represents the mean, whereas the error bars represent the SD across all the session. The underlying data can be found in S1 Data. BOLD, blood oxygen level–dependent; CSF, cerebrospinal fluid; SD, standard deviation. (TIF) [file pbio.3001233.s012.tif]
