# Supplementary material for: Reduced coupling between cerebrospinal fluid flow and global brain activity is linked to Alzheimer disease–related pathology
Source: PLoS Biol. 2021 Jun 1;19(6):e3001233. doi: 10.1371/journal.pbio.3001233 (PMC8168893; doi:10.1371/journal.pbio.3001233)
Supplement: S1 Table — p-Values are derived from 2-sample t test for continuous measures and from Fisher exact test for categorical measures. The underlying data can be found in S1 Data. 24 m follow-up, 24 months follow-up; Aβ florbetapir SUVR, the whole cortical amyloid beta from PET AV45 analysis normalized composite reference region; AD, Alzheimer disease participants; APOE ε4 status (Neg/Pos), not APOE ε4 carrier/APOE ε4 carrier; HC, healthy control; M/F, male/female; M/SD, mean/standard deviation; MCI, mild cognition impairment; MMSE, Mini-Mental State Examination; SMC, significant memory concern. (DOCX) [file pbio.3001233.s013.docx]

**S1 Table. Subject baseline characteristics of the augmented sample.**

| **ADNI(N=154)** | | **A**  **AD(N=29)** | **B**  **MCI(N=62)** | **C**  **SMC(N=18)** | **D**  **HC(N=45)** | ***p*-value** |
| --- | --- | --- | --- | --- | --- | --- |
| Age at baseline (M/SD) | | 73.14 (7.11) | 72.72 (7.17) | 73.17 (5.49) | 74.10 (5.70) | A-B=0.79; A-C=0.99  A-D=0.52; B-C=0.80  B-D=0.29; C-D=0.56 |
| Gender (M/F) | | 14 / 15 | 32/ 30 | 9 / 9 | 19/ 26 | A-B=0.82; A-C=1.00  A-D=0.64; B-C=1.00  B-D=0.43; C-D=0.59 |
| APOE ε4 status (Neg/Pos) (13 N/A) | | 5 / 22 (2 N/A) | 33 / 27  (2 N/A) | 13 / 5 | 25 / 11  (9 N/A) | **A-B=0.002**; **A-C<0.001**  **A-D<0.001**; B-C=0.28  B-D=0.20; C-D=1.00 |
| MMSE (M/SD) | First visit | 22.38 (2.45) | 27.95 (1.95) | 29.17 (0.86) | 28.74 (1.26) | **A-B<0.001**; **A-C<0.001**  **A-D<0.001**; **B-C=0.01**  **B-D=0.04**; C-D=0.20 |
|  | 24m follow-up | 18.63 (5.53) | 27.08 (3.50) | 28.94 (1.00) | 28.79 (1.84) | **A-B<0.001**; **A-C<0.001**  **A-D<0.001**; **B-C=0.03**  **B-D=0.01**; C-D=0.75 |
| Aβ florbetapir SUVR (M/SD) | First visit | 1.08 (0.07) | 0.90 (0.14) | 0.83 (0.10) | 0.81 (0.13) | **A-B=0.001; A-C<0.001**  **A-D<0.001;** B-C=0.08  **B-D=0.004;** C-D=0.48 |
|  | 24m follow-up | 1.11 (0.06) | 0.91 (0.15) | 0.84 (0.12) | 0.82 (0.13) | **A-B<0.001**; **A-C<0.001**  **A-D<0.001**; B-C=0.08  **B-D=0.005**; C-D=0.59 |
